# Fluorinated Analogues of Lepidilines A and C: Synthesis and Screening of Their Anticancer and Antiviral Activity

**DOI:** 10.3390/molecules27113524

**Published:** 2022-05-30

**Authors:** Grzegorz Mlostoń, Mateusz Kowalczyk, Małgorzata Celeda, Marcin Jasiński, Marta Denel-Bobrowska, Agnieszka B. Olejniczak

**Affiliations:** 1 Faculty of Chemistry, University of Lodz, Tamka 12, 91403 Łódź, Poland; mateusz.kowalczyk@edu.uni.lodz.pl (M.K.); malgorzata.celeda@chemia.uni.lodz.pl (M.C.); 2 Institute of Medical Biology, Polish Academy of Sciences, 106 Lodowa St., 93232 Łódź, Poland; mdenel-bobrowska@cbm.pan.pl

**Keywords:** fluorinated heterocycles, lepidilines, natural products, imidazolium salts, sulfur-transfer reactions, anticancer activity, antiviral activity

## Abstract

Starting with fluorinated benzylamines, a series of 2-unsubstituted imidazole *N*-oxides was prepared and subsequently deoxygenated in order to prepare the corresponding imidazoles. The latter were treated with benzyl halides yielding imidazolium salts, which are considered fluorinated analogues of naturally occurring imidazolium alkaloids known as lepidilines A and C. A second series of oxa-lepidiline analogues was obtained by *O*-benzylation of the initially synthetized imidazole *N*-oxides. Both series of imidazolium salts were tested as anticancer and antiviral agents. The obtained results demonstrated that the introduction of a fluorine atom, fluoroalkyl or fluoroalkoxy substituents (F, CF_3_ or OCF_3_) amplifies cytotoxic properties, whereas the cytotoxicity of some fluorinated lepidilines is promising in the context of drug discovery. All studied compounds revealed a lack of antiviral activity against the investigated viruses in the nontoxic concentrations.

## 1. Introduction

**Figure 1:**
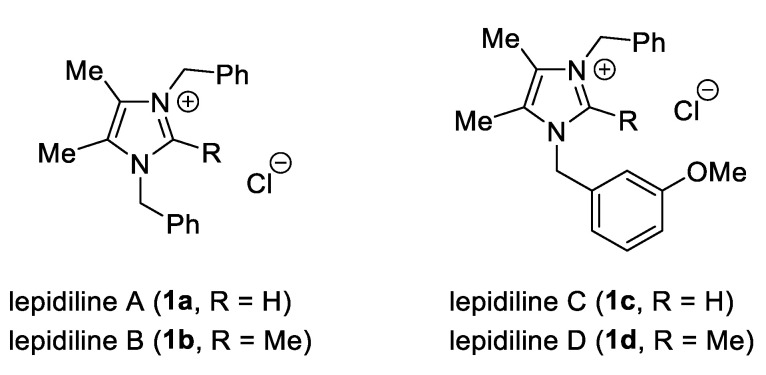
Structures of naturally occurring alkaloids known as lepidilines (A–D).

Dried roots of *Maca* plant (*Lepidium meyenii*) are well-known and widely applied in traditional folk medicine of the South American region for centuries [1]. They have been known to act not only as a natural drug but also as nutritional ingredients. Nowadays, numerous preparations containing powdered *Maca* roots or extracts prepared therefrom are commercially available as food additives and valuable dietary supplements [2,3,4]. Some time ago, imidazolium alkaloids known as lepidilines A–D (Figure 1, **1a**–**1d**) were isolated and identified as significant, biologically active components of *Maca* extracts, and their anticancer activity was reported for the first time in 2003 [5,6]. In a recent publication, resulting from our continuing interest in imidazole chemistry, the synthesis of all lepidilines, as well as their bioactivity, were described. The anticancer activity was checked and compared with the earlier reported results, demonstrating the remarkable cytotoxicity of lepidiline D against the promyelocytic leukemia HL-60 cell lines [7]. In addition, oxa- analogues of lepidilines A and C (alkoxyimidazolium salts) were also synthesized, and the presence of benzyloxy-type substituents was found beneficial in terms of anticancer activity [7,8]. On the other hand, the antiviral activity of isolated lepidilines has not yet been reported. Nevertheless, the antiviral activity of complex *Maca* extracts against the human influenza virus was studied, and remarkable therapeutic effects in the treatment of Flu-A and Flu-B were demonstrated [9]. Prompted by this observation, and taking into account the reported antiviral properties of some imidazole-based compounds [10,11,12,13], we decided to check the antiviral activity of lepidilines A and C and a series of oxy-imidazolium salts that can be considered close structural analogues of these alkaloids.

In general, introducing a fluorine atom or fluoroalkyl groups into the structure of the heterocyclic core of an organic compound substantially increases its bioactivity [14,15]. Therefore, more than 20% of commercially available medicaments constitute hetero-organic compounds functionalized with fluorine-containing substituents [16]. In spite of this fact, fluorine-containing lepidilines are still unknown, and it seemed reasonable to fill this gap. For this reason, we decided to involve a series of fluorinated lepidilines in the present study to check the anticipated beneficial effects of an F atom, as well as CF_3_ and OCF_3_ groups incorporated in their structures, analogous to lepidiline C, at the *m*eta-position of the N(1) benzyl group.

**Figure 2:**
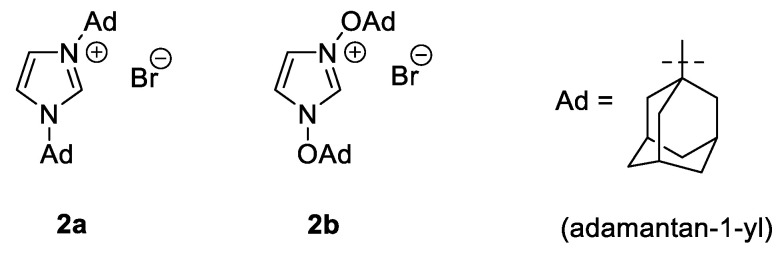
The structure of *N*-adamantanyl-functionalized ‘Arduengo salt’ **2a** and its bis-oxidized analogue, **2b**.

Thus, the main goal of the present study was the synthesis of fluorinated imidazolium salts derived from lepidilines A and C and the examination of their anticancer, as well as antiviral, activity against the selected cell lines (cancer: A549, HepG2, and HeLa and normal: Vero, LLC-MK2, MRC-5, and NCTC clone 929) and model viruses (HSV-1, HCMV, AdV5, HPIV-3, and EMCV), respectively. In addition, taking into account the general importance of 1,3-diadamantyl imidazolium bromide (**2a**, known as ‘Arduengo salt’ [17]), its bis-oxidized analogue **2b** and other structurally related imidazolium salts not only in the chemistry of nucleophilic heterocyclic carbenes [18,19,20] but also in medicinal chemistry [21,22], they were also involved in the present study aimed at the comparison of their antiviral activity with lepidiline analogues (Figure 2).

## 2. Results and Discussion

### 2.1. Chemistry

The preparation of lepidilines **1a** and **1c** was performed following the general procedure described previously employing the respective 2-unsubstituted 4,5-dimethylimidazole *N*-oxides, which, after deoxygenation and quaternization using benzyl chloride, were converted into final products [7,23]. Similarly, Arduengo salt **2a** and its bis-oxy-analogue **2b** were obtained based on published methods via the cyclocondensation of glyoxal with 1-aminoadamantane or adamantyl-1-oxyamine, respectively, in the presence of HBr [24].

**Scheme 1:**
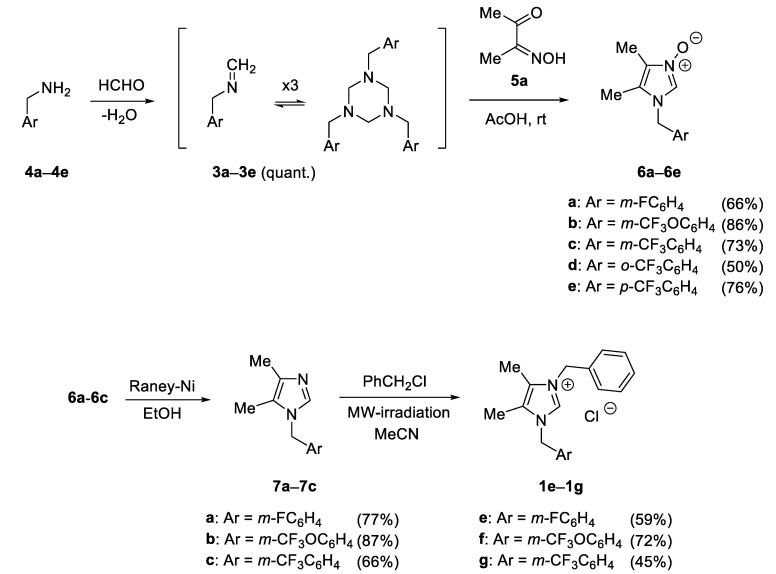
Synthesis of fluorinated lepidiline analogues **1e**–**1g** using 2-unsubstituted imidazole *N*-oxides **6a**–**6c** as the key intermediates.

The synthesis of fluorinated derivatives of lepidilines **1a** and **1c**, i.e., imidazolium salts **1e**–**1g**, started with the preparation of hitherto unknown fluorinated formaldimines **3a**–**3c**, which are available by treatment of the corresponding benzylamines **4a**–**4c** with formaldehyde (Scheme 1). Crude oily products of type **3** were treated with diacetyl monoxime (**5a**) in acetic acid at room temperature, yielding the desired imidazole *N*-oxides **6a**–**6c** in high overall yields (66–86%). In addition, two isomeric benzylamines **4d** and **4e** bearing the CF_3_ group located either at the *ortho* or *para* position of the phenyl ring, respectively, were involved in the study, and the expected imidazole *N*-oxides **6d** and **6e** were obtained (50% and 76% for two steps). In the next step, the *N*-oxides **6a**–**6c** were deoxygenated using freshly prepared Raney-Ni to afford the corresponding 1-benzyl-4,5-dimethylimidazoles **7a**–**7c**. Finally, *N*-benzylation performed with benzyl chloride under microwave irradiation in MeCN led to the desired fluorinated analogues of lepidilines **1e**–**1g** in an acceptable overall yield of 30%, 54%, and 22% (from amines **4**), respectively.

**Scheme 2:**
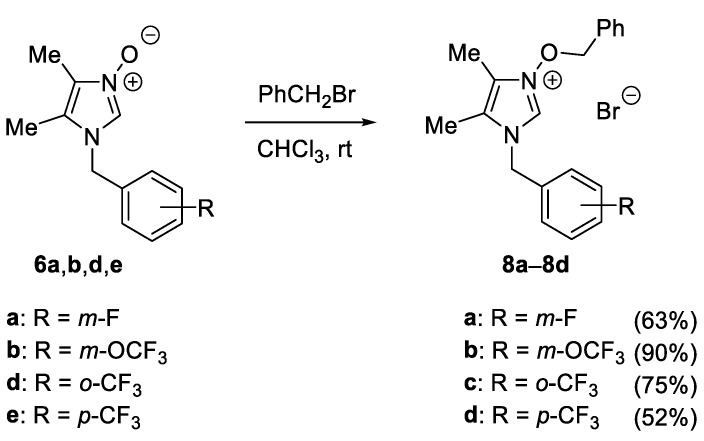
*O*-Benzylation reactions of imidazole *N*-oxides **6a**, **b**, **d**, and **e** with benzyl bromide leading to alkoxyimidazolium salts **8a**–**8d**.

Prompted by our earlier study focused on the preparation and anticancer activity screening of oxidized analogues of lepidilines [7,8], the starting imidazole *N*-oxides **6a**–**6c** were also subjected to benzylation reactions under standard conditions, in CHCl_3_ at room temperature, and in these reactions, no MW activation was necessary to perform *O*-alkylation. In the case of **6a** and **6b**, the expected benzyloxy salts **8a** and **8b** were formed as sole products and isolated as crystalline materials. Analogous results, leading to the formation of imidazolium salts **8c** and **8d** as single products, were obtained using trifluoromethylbenzyl-functionalized imidazole *N*-oxides **6d** and **6e** and benzyl bromide as an alkylating agent (Scheme 2).

**Scheme 3:**
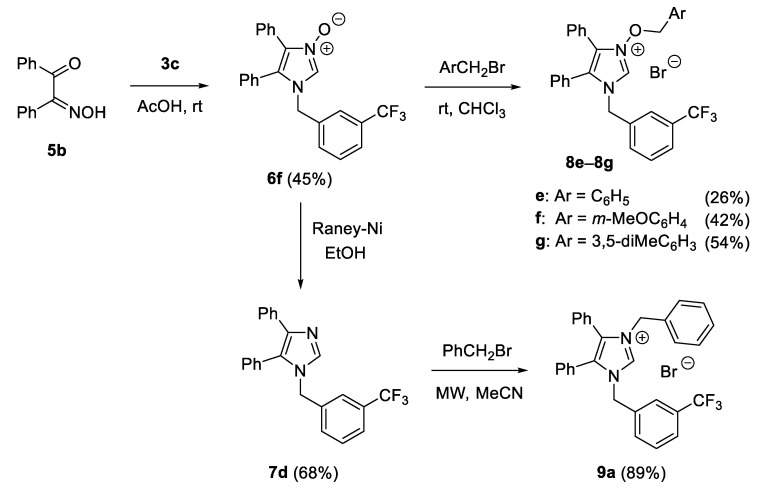
Synthesis of 4,5-diphenyl-functionalized fluorinated alkoxyimidazolium and imidazolium salts **8e**–**8g** and **9a**, respectively.

In extension of the study, collection of lepidiline analogues with a 4,5-dimethyl-substituted imidazolium core was supplemented by 4,5-diphenyl derivative functionalized with the *m*-CF_3_-benzyl group located at N(1) of the imidazolium ring. The key imidazole *N*-oxide **6f** was prepared analogously, starting with benzil monoxime (**5b**) and trimeric formaldimine **3c**. The deoxygenation of **6f** with Raney nickel afforded the desired 1,4,5-trisubstituted imidazole **7d** in 68% yield. Following the general procedure, *N*-benzylation of imidazole **7d** with benzyl bromide provided the expected salt **9a** (Scheme 3) in a 27% overall yield. Similarly, *N*-oxide **6f** was treated with selected benzyl bromides, and the expected benzyloxy-imidazolium salts **8e**–**8g** were obtained as exclusive products.

**Figure 3:**
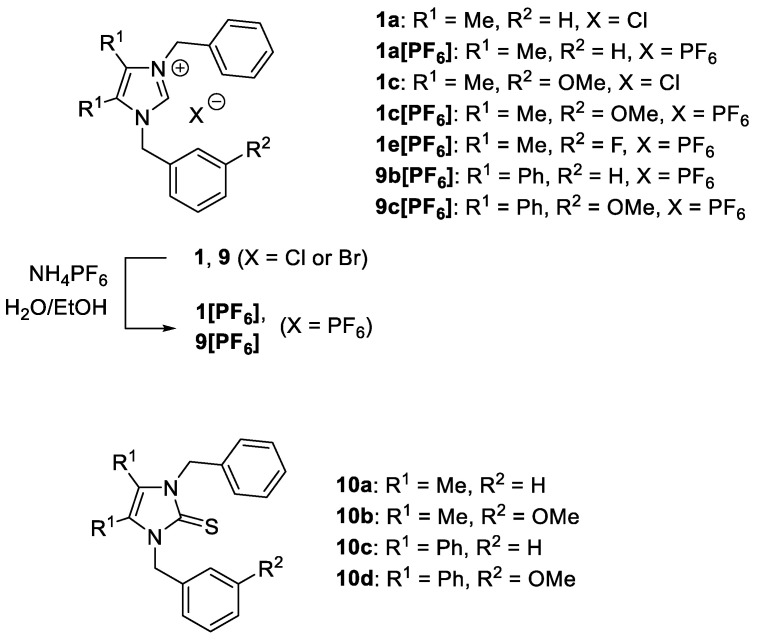
Ion exchange in selected lepidiline-derived imidazolium salts of types **1** and **9** and the structures of imidazole-2-thiones **10** used in this study.

In a recent publication, the anticancer activity of imidazolium salts, considered as lepidiline analogues with no fluorinated benzyl substituents at N(1), was reported [7]. For comparison of the antiviral activity of both series of lepidiline analogues, i.e., fluorinated and non-fluorinated representatives, they were also involved in the present study. In order to check the influence of the counterion present in imidazolium salts **1** and **9** on the biological activity, selected chlorides were converted into the corresponding hexafluorophosphates **1[PF_6_]** and **9[PF_6_]** by counterion exchange via treatment with ammonium hexafluorophosphate in water/ethanol solution (Figure 3). Furthermore, it is well-known that imidazolium salts are the perfect substrates for the preparation of the corresponding imidazole-2-thiones via nucleophilic carbenes (NHCs) as the in situ generated intermediates [7,18,25,26]. For that reason, some imidazole-2-thiones (**10**) shown in Figure 3 were also involved in the study focused on antiviral and anticancer activity screening presented in this work (see Supplementary Materials).

### 2.2. In Vitro Cytotoxicity on Cancer and Normal Cell Lines

Despite the continuous development of modern medicine, finding an effective cure for neoplastic diseases, especially those diagnosed in an advanced stage, is still a challenge. Screening studies for potential anticancer agents is a crucial step in cancer drug discovery. An ideal situation is when the drug can kill the cancer cells but, at the same time, not affect the normal cells [27]. Therefore, it is advantageous to include normal (noncancer) cells in research on the cytotoxicity of potential drugs.

The initial step of our studies was to test cytotoxic properties of the series compounds **1a**, **1c**, **1a[PF_6_]**, **1c[PF_6_]**, **9b[PF_6_]**, **9c[PF_6_]**, **2a**, **2b**, and **10a**–**10d** during a screening assay at 10 µM on Cercopithecus aethiops normal kidney cells (Vero), Macaca mulatta normal kidney cells (LLC-MK2), Human lung normal fibroblasts (MRC-5), Mus musculus normal subcutaneous connective tissue cells (NCTC clone 929), and Human cervix adenocarcinoma cells (HeLa). Compounds demonstrating cell viability ≥ 50% (in both cytotoxicity and antiviral activity studies mentioned below) were selected for further, extended studies resulting in CC_50_ (50% cytotoxic concentration, the parameter used for cytotoxicity results) and IC_50_ (50% inhibitory concentrations, the parameter used for antiviral activity results) (Tables S1–S4, Supplementary Materials).

Screening cytotoxicity studies revealed that the most promising results were observed for compounds **1a**; **1c; 1a[PF_6_]**; **1c[PF_6_]** (LLC-MK2 cell line); **10a** (LLC-MK2, NCTC clone 929, and HeLa cell lines); and **10d** (HeLa cell line). Compound **10a** showed the highest cytotoxicity (CC_50_ < 20 µM) on the LLC-MK2 and HeLa cell lines, whereas compounds: **1a**, **1c**, **1a[PF_6_],** and **1c[PF_6_]** were cytotoxic in the concentration of CC_50_ < 80 µM on LLC-MK2 cells. Compound **10d** was nontoxic against the tested HeLa cells (CC_50_ < 400 µM) (Tables S1 and S2, Supplementary Materials).

The fluorine atom is a key part of the medicinal chemist’s repertoire of substitutions used to modulate all aspects of molecular properties, including potency, physical chemistry, and pharmacokinetics [15,28]. Fluorinated compounds are an important class of anticancer and antiviral drugs [29,30].

The in vitro cytotoxic activities of the target compounds **1e**–**1g**, **1e[PF_6_]**, **8a**–**8d**, and **9a** were investigated in two types of human cell lines—four normal cell lines: Vero, LLC-MK2, MRC-5, and NCTC clone 929, as well as three cancer cell lines: HeLa, Human lung carcinoma cells (A549), and Human hepatocellular carcinoma cells (HepG2). Cytotoxicity of the investigated compounds was established by the measurement of 50% inhibition of cell growth by the MTT assay and expressed as the CC_50_ parameter (50% cytotoxic concentration). All results are presented in Table 1.

Individual cell lines were characterized by different sensitivities to the tested compounds. The HeLa cell line showed the highest sensitivity among all the tested cell lines, but for some of the investigated compounds, the results obtained for A549 were similar to those observed in HeLa cells. The lowest sensitivity towards the tested compounds was obtained for the HepG2 cells.

Generally, fluorinated lepidilines **1e**–**1g**, **1e[PF_6_]**, and **9a** were found to be the most cytotoxic against the HeLa cell line, with CC_50_ values significantly below 1 µM. 4,5-Diphenyl derivative **9a** was the most cytotoxic at a concentration as low as 0.019 µM. Its analogues bearing dimethyl groups attached at positions 4 and 5 (**1e**–**1g** and **1e[PF_6_**]) were less active (CC_50_ = 0.039–0.080 µM), and for compound **1g**, this activity was almost three times lower compared to **9a**. Recently, we published that 4,5-diphenyl analogues of lepidilines A, C, and D showed also increased cytotoxicity against the MCF-7 cell line compared to the corresponding lepidilines bearing methyl groups at C(4) and C(5) of the imidazole ring [7]. The presence of an F atom in compound **1e** or OCF_3_ group in compound **1f** resulted in the increase of their activity for the HeLa cell line compared to **1g**. Interestingly, the replacement of Cl^−^ (compound **1e**) with PF_6_^−^ (compound **1e[PF_6_]**) resulted in a two-fold decrease in the cytotoxicity. Comparison activities of fluorinated lepidilines in the series containing 4,5-dimethyl groups (**1e**–**1g**, **1e[PF_6_]**, and **8a**–**8d**) revealed that oxidized analogues **8a**–**8d** were much less active against HeLa cells, with CC_50_ values in the range 5.500–20.000 µM, which were two (**8b**, **8d**) or three (**8a**, **8c**) orders of magnitude lower than unoxidized **1e**–**1g** and **1e[PF_6_]**.

Lepidiline **9a** also showed high cytotoxicity in the A549 cell line, but other non-oxidized lepidilines: **1e**–**1g** and **1e[PF_6_]** were less active on the same cell line. It should be noted that all oxidized lepidilines **8a**–**8d** revealed better cytotoxic activity against the A549 cell line (CC_50_ = 5.070–7.500 µM) than against the HeLa cell line (CC_50_ = 5.500–20.000 µM). In the experiments performed on HepG2 cells, the CC_50_ values indicated that all the tested lepidilines, except for **9a** (CC_50_ = 6.900 µM), were not toxic (CC_50_ > 90 µM). The most cytotoxic against the selected cancer cell lines, lepidilines **1e**–**1g,** and **1e[PF_6_]** were rather nontoxic against normal cell lines—MRC-5 (CC_50_ = 36.667–149.667 µM), NCNT clone 929 (CC_50_ = 30.000–148.333 µM), LLC-MK2 (CC_50_ = 53.500–150.333 µM), and Vero (CC_50_ = 22.667–56.333 µM), with the exception of **1g**, which is toxic against Velo cells. Compound **9a**, cytotoxic to all the tested cancer cell lines, is also toxic against MRC-5, LLC-MK2, and Vero (CC_50_ = 0.040–0.347 µM); it is less toxic to NCNT 929 (CC_50_ = 3.750 µM). In the experiments performed on normal cells, the CC_50_ values for almost all oxidized lepidilines **8a**–**8d** were > 50 µM, which indicates a large safety margin for these compounds.

### 2.3. In Vitro Antiviral Activity

The search for compounds with antiviral activity among the natural compounds and their modified analogues is a rapidly developing direction in pharmaceutical chemistry.

Screening of the antiviral activity of compounds **1a**, **1c**, **1a[PF_6_]**, **1c[PF_6_]**, **9b[PF_6_]**, **9c[PF_6_]**, **2a**, **2b**, and **10a**–**10d** against viruses: Human herpesvirus 1 (HSV-1), Human herpesvirus 5 (HCMV), Human adenovirus 5 (AdV5), Human parainfluenza virus type 3 (HPIV-3), and Encephalomyocarditis virus (EMCV) demonstrated that all the compounds revealed a lack of antiviral activity against all investigated viruses in the nontoxic concentrations (Tables S3 and S4, Supplementary Materials).

The antiviral activity of **1e**–**1g**, **1e[PF_6_]**, **8a**–**8d**, and **9a** was also evaluated against the same viruses (HSV-1, HCMV, AdV5, HPIV-3, and EMCV). The antiviral activity results were shown as an IC_50_ parameter (50% inhibitory concentrations).

According to the results collected in Table 2, we can conclude that all investigated compounds revealed a lack of antiviral activity against viruses: HSV-1, HPIV-3, AdV5, EMCV, and HCMV in the nontoxic concentrations.

## 3. Conclusions

A series of fluorinated imidazolium (compounds of type **1** and **9**) and their oxa analogues (compounds of type **8**), considered as close structural analogues of naturally occurring imidazolium alkaloids, known as lepidilines A and C, was prepared, and their anticancer, as well as antiviral activity, was examined. The target products were prepared via a straightforward, three-step protocol starting with benzylamines functionalized either with the F atom or with CF_3_ (or OCF_3_) groups. The presented study demonstrated, once more, a high utility of 2-unsubstituted imidazole *N*-oxides as key intermediates for the synthesis of polyfunctionalized imidazole derivatives.

The obtained new imidazolium salts demonstrated various cytotoxicity levels towards the tested normal and cancer cell lines. Notably, the introduction of fluorinated benzyl substituents resulted, in some cases, in a remarkable increase of bioactivity. For example, fluorinated analogues of lepidilines A and C, i.e., compounds **1e**–**1g** and **1e[PF_6_]**, **8a**–**8d**, and **9a** were the most active against the HeLa or A549 cell lines. Their cytotoxicity was significantly higher in comparison with natural lepidiline A against the HeLa cell line. Remarkably, the most cytotoxic compound **9a** was also toxic against normal cell lines. In contrast, derivatives **1e**–**f** and **1e[PF_6_]**, **8a**–**8d** were found to be rather nontoxic in the normal cell lines. All investigated compounds revealed no antiviral activity against HSV-1, HPIV-3, AdV5, EMCV, and HCMV in the range of nontoxic concentrations. The presented results confirmed the importance of fluorinated substituents for tuning the biological activity of organic compounds [28,29], including some naturally occurring imidazolium salts, such as lepidilines A and C, and their 4,5-diphenyl analogues.

The results obtained in the testing of the antiviral properties of lepidilines A and C, as well as their fluorinated analogues, suggest that the earlier reported antiviral activity of *Maca* extract [9] results rather from the presence of other compounds, e.g., indole derivatives or complex isothiocyanates, which were also identified as its components [31,32,33].

## 4. Materials and Methods

### 4.1. General Synthetic Procedures

Commercial chemicals and solvents were used as received. If not stated otherwise, products were purified by filtration through a short silica gel plug (200–400 mesh) by using freshly distilled solvents as eluents or by recrystallization. Melting points were determined in capillaries with an Aldrich Melt-Temp II, and they are uncorrected. NMR spectra were taken with a Bruker AVIII spectrometer (^1^H NMR (600 MHz), ^13^C NMR (151 MHz), and ^19^F NMR (565 MHz); chemical shifts are relative to the residual undeuterated solvent peaks (CDCl_3_: ^1^H NMR δ = 7.26, ^13^C NMR δ = 77.16 [34]) or to the external standard (CFCl_3_: ^19^F NMR δ = 0.00). IR spectra were measured with an Agilent Cary 630 FTIR spectrometer neat. Mass spectra (ESI) were obtained with a Varian 500-MS LC Ion Trap. Elemental analyses were obtained with a Vario EL III (Elementar Analysensysteme GmbH) instrument. Starting α-hydroxyiminoketones **5a** [35] and **5b** [36] were prepared following the general literature protocols.

#### 4.1.1. Synthesis of Imidazolium Chlorides **1e**–**g** and **9a**

To a deoxygenated solution of imidazole **7** (1.0 mmol) in MeCN (2.0 mL) was added benzyl halide (1.2 mmol), and the resulting mixture was MW-irradiated at 110 °C in a closed vessel until the starting imidazole was fully consumed (TLC monitoring, typically ca. 45 min). After the solvent was removed under reduced pressure, the crude product was washed with several portions of dry Et_2_O (5 × 5 mL), and the solid imidazolium chloride was recrystallized from a CH_2_Cl_2_/hexane mixture.

*1-Benzyl-3-(3-fluorobenzyl)-4,5-dimethylimidazolium chloride* (**1e**): 195 mg (59%), pale yellow crystals, m.p. 190–192 °C. ^1^H NMR (600 MHz, CDCl_3_): *δ* 2.05 (s_br_, 6 H, 2 Me), 5.47, 5.59 (2 s, 2 H each, 2 CH_2_), 6.95–7.00, 7.10–7.12, 7.26–7.34 (3 m, 2 H, 1 H, 6 H), 11.18 (s, 1 H, C(2)H). ^13^C NMR (151 MHz, CDCl_3_): *δ* 8.87, 8.88, 50.4 (d, ^4^*J*_C__,F_ = 1.4 Hz, CH_2_), 51.2, 114.8 (d, ^2^*J*_C__,F_ = 22.4 Hz, CH), 116.0 (d, ^2^*J*_C__,F_ = 21.1 Hz, CH), 123.6 (d, ^4^*J*_C__,F_ = 2.9 Hz, CH), 127.2, 127.3, 127.8, 129.0, 129.4, 131.1 (d, ^3^*J*_C__,F_ = 8.2 Hz, CH), 133.1, 135.8 (d, ^3^*J*_C__,F_ = 7.2 Hz, *i*-C), 137.6(br)*, 163.0 (d, ^1^*J*_C__–F_ = 248.3 Hz, *i*-C); broadened signal due to partial H/D exchange at C(2). ^19^F NMR (565 MHz, CDCl_3_): *δ* −111.2 (m_c_, CF). ESI-MS (*m*/*z*): 295.4 (100, [*M* − Cl]^+^). HRMS (ESI-TOF) *m/z*: [*M* − Cl]^+^ calcd for C_19_H_20_FN_2_ 295.1611, found 295.1612. 

*1-Benzyl-4,5-dimethyl-3-[3-(trifluoromethoxy)benzyl]imidazolium chloride* (**1f**): 285 mg (72%), colorless crystals, m.p. 108–110 °C. ^1^H NMR (600 MHz, CDCl_3_): *δ* 2.05 (s_br_, 6 H, 2 Me), 5.49, 5.65 (2 s, 2 H each, 2 CH_2_), 7.07 (m_c_, 1 H), 7.13–7.16, 7.24–7.41 (2 m, 1 H, 7 H), 10.94 (s, 1 H, C(2)H). ^13^C NMR (151 MHz, CDCl_3_): *δ* 8.8, 8.9, 50.3, 51.2, 120.2, 120.4 (q, ^1^*J*_C__–F_ = 258.0 Hz, OCF_3_), 121.2, 126.4, 127.2, 127.4, 127.7, 129.0, 129.4, 131.1, 133.1, 135.9, 137.6(br), 149.6. ^19^F NMR (565 MHz, CDCl_3_): *δ* −57.8 (s, OCF_3_). IR (neat): *ν* 1554, 1260, 1208, 1171, 758, 701 cm^−1^. ESI-MS (*m*/*z*): 361.4 (100, [*M* – Cl]^+^). HRMS (ESI-TOF) *m/z*: [*M* − Cl]^+^ calcd for C_20_H_20_F_3_N_2_O 361.1528, found 361.1531.

*1-Benzyl-4,5-dimethyl-3-[3-(trifluoromethyl)benzyl]imidazolium chloride* (**1g**): 171 mg (45%), colorless crystals, m.p. 120–122 °C. ^1^H NMR (600 MHz, CDCl_3_): *δ* 2.05 (s_br_, 6 H, 2 Me), 5.49, 5.71 (2 s, 2 H each, 2 CH_2_), 7.25–7.34 (m, 5 H), 7.45 (m_c_, 1 H), 7.47–7.50, 7.53–7.55, 7.63–7.65 (3 m, 1 H each), 10.96 (s, 1 H, C(2)H). ^13^C NMR (151 MHz, CDCl_3_): *δ* 8.87, 8.88, 50.4, 51.2, 123.7 (q, ^1^*J*_C__–F_ = 272.5 Hz, CF_3_), 124.2 (q, ^3^*J*_CF_ = 3.6 Hz, CH), 125.8 (q, ^3^*J*_C__,F_ = 3.6 Hz, CH), 127.1, 127.4, 127.7, 129.0, 129.4, 130.2, 131.5 (q, ^2^*J*_C__,F_ = 32.6 Hz, *i*-C), 131.6, 133.1, 134.7, 137.5(br). ^19^F NMR (565 MHz, CDCl_3_): *δ* −62.6 (s, CF_3_). IR (neat): *ν* 1551, 1454, 1327, 1163, 1115, 1074, 701 cm^−1^. ESI-MS (*m*/*z*): 345.4 (100, [*M*−Cl]^+^). HRMS (ESI-TOF) *m/z*: [*M* − Cl]^+^ calcd for C_20_H_20_F_3_N_2_ 345.1579, found 345.1577.

*1-Benzyl-4,5-diphenyl-3-[3-(trifluoromethyl)benzyl]imidazolium bromide* (**9a**): 488 mg (89%), colorless crystals, m.p. 216–217 °C. ^1^H NMR (600 MHz, CDCl_3_): *δ* 5.47, 5.68 (2 s, 2 H each, 2 CH_2_), 6.82 (m_c_, 1 H), 7.03–7.11, 7.20–7.31, 7.37–7.47, 7.77–7.80 (4 m, 6 H, 7 H, 4 H, 1 H), 11.38 (s, 1 H, C(2)H). ^13^C NMR (151 MHz, CDCl_3_): *δ* 51.0, 51.7, 123.5 (q, ^1^*J*_C__–F_ = 272.5 Hz, CF_3_), 124.5, 124.6, 125.3 (q, ^3^*J*_C__,F_ = 3.7 Hz, CH), 125.8 (q, ^3^*J*_C__,F_ = 3.7 Hz, CH), 128.5, 129.1(br), 129.16, 129.21, 129.3, 130.1, 130.6, 130.7, 130.75, 130.81, 130.9 (q, ^2^*J*_C__−F_ = 32.6 Hz, *i*-C), 132.2, 132.5, 132.7(br), 133.2, 134.3, 137.8(br). ^19^F NMR (565 MHz, CDCl_3_): *δ* −62.8 (s, CF_3_). IR (neat): *ν* 1554, 1450, 1327, 1170, 1122, 1074, 697 cm^−1^. ESI-MS (*m*/*z*): 469.6 (100, [*M* – Br]^+^). Anal. calcd for C_30_H_24_BrF_3_N_2_ (548.1): C 65.58, H 4.40, N 5.10; found: C 65.36, H 4.40, N 4.90.

#### 4.1.2. Synthesis of Imidazolium Hexafluorophosphate **1e[PF_6_]**

To a solution of imidazolium chloride **1e** (89 mg, 0.27 mmol) in EtOH (2.0 mL) was added dropwise a solution of NH_4_PF_6_ (47 mg, 0.29 mmol) in H_2_O (1.0 mL), and the mixture was stirred for 30 min. The precipitate of the crude product was isolated, washed with dry Et_2_O (4 × 4 mL), and recrystallized from a CH_2_Cl_2_/*i*-Pr_2_O mixture by slow evaporation of the solvents at room temperature.

*1-Benzyl-3-(3-fluorobenzyl)-4,5-dimethylimidazolium hexafluorophosphate* (**1e[PF_6_]**): 53 mg (45%), colorless crystals, m.p. 110–112 °C. ^1^H NMR (600 MHz, CDCl_3_): *δ* 2.04, 2.05 (2 s, 3 H each, 2 Me), 5.21, 5.22 (2 s, 2 H each, 2 CH_2_), 6.89–6.91, 6.96–7.00, 7.19–7.21, 7.28–7.35 (4 m, 1 H, 2 H, 2 H, 4 H), 8.63 (s, 1 H, C(2)H). ^13^C NMR (151 MHz, CDCl_3_): *δ* 8.57, 8.63, 50.5 (d, ^4^*J*_C__,F_ = 2.1 Hz, CH_2_), 51.3, 114.8 (d, ^2^*J*_C__,F_ = 22.6 Hz, CH), 116.2 (d, ^2^*J*_C__,F_ = 20.9 Hz, CH), 123.5 (d, ^4^*J*_C__,F_ = 3.1 Hz, CH), 127.8, 128.1, 128.2, 129.2, 129.6, 131.4 (d, ^3^*J*_C__,F_ = 8.3 Hz, CH), 132.6, 134.7, 135.2 (d, ^3^*J*_C__,F_ = 7.4 Hz, *i*-C), 163.1 (d, ^1^*J*_C__–F_ = 248.1 Hz, *i*-C). ^19^F NMR (565 MHz, CDCl_3_): *δ* −72.0 (d, ^1^*J*_P__–F_ = 712.8 Hz, PF_6_), −111.1 (m_c_, CF). IR (neat): *ν* 1586, 1450, 1353, 1252, 1208, 824, 738 cm^−1^. ESI-MS (*m*/*z*): 295.4 (100, [*M* − PF_6_]^+^).

#### 4.1.3. Synthesis of Alkoxy-Imidazolium Bromides **8**

To a solution of corresponding imidazole *N*-oxide **6** (1.0 mmol) in CHCl_3_ (3.0 mL) was added excess alkyl bromide (1.1 mmol), and the resulting mixture was stirred at room temperature until the starting *N*-oxide was fully consumed (TLC monitoring, SiO_2_ EtOAc/MeOH 95:5). After the solvent was removed under reduced pressure, the resulting crude product was triturated with Et_2_O (4 × 10 mL) and dried under vacuum. Crude products were recrystallized from diisopropyl ether/dichloromethane mixtures by slow evaporation of the solvents at room temperature.

*3-Benzyloxy-1-(3-fluorobenzyl)-4,5-dimethylimidazolium bromide* (**8a**): 246 mg (63%), beige solid, m.p. 124–126 °C. ^1^H NMR (600 MHz, CDCl_3_): *δ* 1.94, 2.06 (2 s, 3 H each, 2 Me), 5.56, 5.65 (2 s, 2 H each, 2 CH_2_), 6.91–6.94, 7.00–7.04, 7.13–7.15, 7.31–7.35, 7.37–7.44, 7.49–7.51 (6 m, 1 H, 1 H, 1 H, 1 H, 3 H, 2 H), 11.08 (s, 1 H, C(2)H). ^13^C NMR (151 MHz, CDCl_3_): *δ* 7.3, 9.1, 50.6 (d, ^4^*J*_C__,F_ = 2.0 Hz, CH_2_), 84.0, 114.9 (d, ^2^*J*_C__,F_ = 22.5 Hz, CH), 116.1 (d, ^2^*J*_C__,F_ = 20.9 Hz, CH), 123.9 (d, ^4^*J*_C__,F_ = 3.0 Hz, CH), 124.4, 125.1, 129.2, 130.6, 130.7, 131.1 (d, ^3^*J*_C__,F_ = 8.3 Hz, CH), 131.5, 132.6(br), 135.5 (d, ^3^*J*_C__,F_ = 7.3 Hz, *i*-C), 163.0 (d, ^1^*J*_C__–F_ = 248.4 Hz, *i*-C). ^19^F NMR (565 MHz, CDCl_3_): *δ* −111.0 (m_c_, CF). IR (neat): *ν* 1592, 1454, 1245, 1141, 947, 917 cm^−1^. ESI-MS (*m*/*z*): 311.4 (100, [*M* – Br]^+^). Anal. calcd for C_19_H_20_BrFN_2_O (390.1): C 58.32, H 5.15, N 7.16; found: C 58.21, H 5.14, N 6.87.

*3-Benzyloxy-4,5-dimethyl-1-[3-(trifluoromethoxy)benzyl]imidazolium bromide* (**8b**): 410 mg (90%), pale yellow solid, m.p. 108–110 °C. ^1^H NMR (600 MHz, CDCl_3_): *δ* 1.92, 2.06 (2 s, 3 H each, 2 Me), 5.53, 5.70 (2 s, 2 H each, 2 CH_2_), 7.04 (m_c_, 1 H), 7.16–7.19, 7.32–7.42, 7.46–7.48 (3 m, 1 H, 5 H, 2 H), 11.07 (s, 1 H, C(2)H). ^13^C NMR (151 MHz, CDCl_3_): *δ* 7.3, 9.1, 50.6, 84.1, 120.3 (q, ^1^*J*_C__–F_ = 258.0 Hz, OCF_3_), 120.4, 121.2, 124.4, 125.2, 126.7, 129.2, 130.5, 130.7, 131.0, 131.5, 132.6(br), 135.4, 149.6 (q, ^3^*J*_C__,F_ = 1.6 Hz, *i*-C). ^19^F NMR (565 MHz, CDCl_3_): *δ* −57.8 (s, OCF_3_). IR (neat): *ν* 1446, 1238, 1215, 1148, 910, 707 cm^−1^. ESI-MS (*m*/*z*): 377.5 (100, [*M* – Br]^+^). Anal. calcd for C_20_H_20_BrF_3_N_2_O_2_ (456.1): C 52.53, H 4.41, N 6.13; found: C 52.39, H 4.42, N 5.85.

*3-Benzyloxy-4,5-dimethyl-1-(2-(trifluoromethyl)benzyl)imidazolium bromide* (**8c**): 330 mg (75%), colorless solid, m.p. 123–125 °C. ^1^H NMR (600 MHz, CDCl_3_): *δ* 1.95, 2.04 (2 s, 3 H each, 2 Me), 5.65, 5.69 (2 s, 2 H each, 2 CH_2_), 7.38–7.43, 7.50–7.58, 7.61–7.64, 7.71–7.73 (4 m, 3 H, 4 H, 1 H, 1 H), 10.42 (s, 1 H, C(2)H). ^13^C NMR (151 MHz, CDCl_3_): *δ* 7.3, 8.7, 47.8* (q, *J*_C__,F_ = 2.8 Hz, CH_2_), 84.2, 124.0 (q, ^1^*J*_C__–F_ = 273.8 Hz, CF_3_), 124.2, 125.2, 126.8 (q, ^3^*J*_C__,F_ = 5.6 Hz, CH), 128.2 (q, ^2^*J*_C__,F_ = 30.9 Hz, *i*-C), 129.2, 129.6, 130.5, 130.6(br), 130.87, 130.92, 131.9, 133.2(br), 133.4; *through-space C–F coupling. ^19^F NMR (565 MHz, CDCl_3_): *δ* −59.7 (s, CF_3_). IR (neat): *ν* 1446, 1312, 1170, 1103, 1040, 951 cm^−1^. ESI-MS (*m*/*z*): 361.7 (100, [*M* – Br]^+^). HRMS (ESI-TOF) *m/z*: [*M* – Br]^+^ calcd for C_20_H_20_F_3_N_2_O 361.1528, found 361.1529.

*3-Benzyloxy-4,5-dimethyl-1-[4-(trifluoromethyl)benzyl]imidazolium bromide* (**8d**): 229 mg (52%), colorless solid, m.p. 180–182 °C (decomp.). ^1^H NMR (600 MHz, CDCl_3_): *δ* 1.94, 2.07 (2 s, 3 H each, 2 Me), 5.55, 5.76 (2 s, 2 H each, 2 CH_2_), 7.37–7.44, 7.47–7.50, 7.58–7.60 (3 m, 3 H, 4 H, 2 H), 11.10 (s, 1 H, C(2)H). ^13^C NMR (151 MHz, CDCl_3_): *δ* 7.3, 9.1, 50.7, 84.1, 123.8 (q, ^1^*J*_C__–F_ = 272.3 Hz, CF_3_), 124.4, 125.2, 126.3 (q, ^3^*J*_C__,F_ = 3.7 Hz, 2 CH), 128.7, 129.3, 130.6, 130.7, 131.3 (q, ^2^*J*_C__,F_ = 32.7 Hz, *i*-C), 131.5, 132.8(br), 137.1. ^19^F NMR (565 MHz, CDCl_3_): *δ* −62.8 (s, CF_3_). IR (neat): *ν* 1420, 1320, 1141, 1111, 1066, 910, 772 cm^−1^. ESI-MS (*m*/*z*): 361.4 (100, [*M* – Br]^+^). Anal. calcd for C_20_H_20_BrF_3_N_2_O (440.1): C 54.44, H 4.57, N 6.35; found: C 54.32, H 4.70, N 6.44.

*3-Benzyloxy-4,5-diphenyl-1-[3-(trifluoromethyl)benzyl]imidazolium bromide* (**8e**): 147 mg (26%), colorless solid, m.p. 107–110 °C. ^1^H NMR (600 MHz, CDCl_3_): *δ* 5.38, 5.70 (2 s, 2 H each, 2 CH_2_), 6.85 (m_c_, 1 H), 7.12–7.23, 7.28–7.32, 7.38–7.46, 7.50–7.53, 7.72–7.74 (5 m, 8 H, 3 H, 4 H, 2 H, 1 H), 11.44 (s, 1 H, C(2)H). ^13^C NMR (151 MHz, CDCl_3_): *δ* 51.0, 84.6, 123.5 (q, ^1^*J*_C__–F_ = 272.4 Hz, CF_3_), 122.9, 124.3, 125.5 (q, ^3^*J*_C__,F_ = 3.6 Hz, CH), 125.9 (q, ^3^*J*_C__,F_ = 3.5 Hz, CH), 128.8, 128.87, 128.94, 129.2, 129.6, 129.7, 130.0, 130.2, 130.4, 130.7, 130.9, 131.0 (q, ^2^*J*_C__,F_ = 32.6 Hz, *i*-C), 131.1, 131.2, 132.0 (br), 134.1(br), 134.2. ^19^F NMR (565 MHz, CDCl_3_): *δ* −62.7 (s, CF_3_). IR (neat): *ν* 1539, 1457, 1316, 1159, 1122, 1077, 887, 757 cm^−1^. ESI-MS (*m*/*z*): 485.6 (100, [*M* – Br]^+^). Anal. calcd for C_30_H_24_BrF_3_N_2_O (564.1): C 63.73, H 4.28, N 4.95; found: C 63.57, H 4.16, N 4.69.

*3-[(3-Methoxy)benzyloxy]-4,5-diphenyl-1-[3-(trifluoromethyl)benzyl]imidazolium bromide* (**8f**): 250 mg (42%), colorless solid, m.p. 132–134 °C. ^1^H NMR (600 MHz, CDCl_3_): *δ* 3.71 (s, 3 H, Ome), 5.37, 5.71 (2 s, 2 H each, 2 CH_2_), 6.75–6.79, 6.85–6.87, 7.10–7.16, 7.29–7.32, 7.38–7.48, 7.51–7.54, 7.75–7.78 (7 m, 2 H, 2 H, 5 H, 2 H, 4 H, 2 H, 1 H), 11.52 (s, 1 H, C(2)H). ^13^C NMR (151 MHz, CDCl_3_): *δ* 50.9, 55.5, 84.7, 115.2, 116.9, 122.9, 123.0, 123.6 (q, ^1^*J*_C__–F_ = 272.5 Hz, CF_3_), 124.3, 125.6 (q, ^3^*J*_C__,F_ = 3.5 Hz, CH), 126.0 (q, ^3^*J*_C__,F_ = 3.6 Hz, CH), 128.8, 129.0, 129.66, 129.72, 129.9, 130.1, 130.4, 131.06 (q, ^2^*J*_C__,F_ = 32.5 Hz, *i*-C), 131.08, 131.3, 132.2, 132.9(br), 134.1(br), 134.2, 159.9. ^19^F NMR (565 MHz, CDCl_3_): *δ* −62.8 (s, CF_3_). IR (neat): *ν* 1599, 1491, 1446, 1327, 1267, 1167, 1115, 1074, 928 cm^−1^. ESI-MS (*m*/*z*): 515.6 (100, [*M* – Br]^+^). Anal. calcd for C_31_H_26_BrF_3_N_2_O_2_ (594.1): C 62.53, H 4.40, N 4.70; found: C 62.55, H 4.31, N 4.52.

*3-[(3,5-Dimethyl)benzyloxy]-4,5-diphenyl-1-[3-(trifluoromethyl)benzyl]imidazolium bromide* (**8g**): 320 mg (54%), colorless solid, m.p. 156–158 °C. ^1^H NMR (600 MHz, CDCl_3_): *δ* 2.18 (s, 6 H, 2 Me), 5.29, 5.73 (2 s, 2 H each, 2 CH_2_), 6.73, 6.84, 6.93 (3 m_c_, 2 H, 1 H, 1 H), 7.09–7.15, 7.28–7.31, 7.38–7.49, 7.52–7.55, 7.81–7.83 (5 m, 4 H, 2 H, 4 H, 2 H, 1 H), 11.55 (s, 1 H, C(2)H). ^13^C NMR (151 MHz, CDCl_3_): *δ* 21.1, 50.9, 85.1, 122.9, 123.6 (q, ^1^*J*_C__–F_ = 272.5 Hz, CF_3_), 124.4, 125.7 (q, ^3^*J*_C__,F_ = 3.8 Hz, CH), 126.0 (q, ^3^*J*_C__,F_ = 3.4 Hz, CH), 128.4, 128.7, 128.9, 129.4, 129.68, 129.72, 130.1, 130.3, 130.7, 131.0 (q, ^2^*J*_C__,F_ = 32.6 Hz, *i*-C), 131.1, 131.3, 132.0, 133.0, 134.1(br), 134.3, 138.5. ^19^F NMR (565 MHz, CDCl_3_): *δ* −62.8 (s, CF_3_). IR (neat): *ν* 1543, 1446, 1327, 1167, 1118, 1074, 760 cm^−1^. ESI-MS (*m*/*z*): 513.5 (100, [*M* – Br]^+^). Anal. calcd for C_32_H_28_BrF_3_N_2_O (592.1): C 64.76, H 4.76, N 4.72; found: C 64.69, H 4.54, N 4.39.

### 4.2. In Vitro Cytotoxicity and Antiviral Activity

#### 4.2.1. Cytotoxicity Screening of Compounds at a Concentration of 10 µM

Cytotoxic properties of compounds **1a**, **1c**, **1a[PF_6_]**, **1c[PF_6_]**, **9b[PF_6_]**, **9c[PF_6_]**, **10a**–**10d**, **2a**, and **2b** were assessed on Vero (CCL-81, Cercopithecus aethiops normal kidney cells), LLC-MK2 (CCL-7, Macaca mulatta normal kidney cells), MRC-5 (CCL-171, Human lung normal fibroblasts), NCTC clone 929 (CCL-1, Mus musculus normal subcutaneous connective tissue cells), and HeLa (CCL-2, Human cervix adenocarcinoma cells) cell lines. Cell lines were purchased from the American Type Culture Collection (ATCC, Manassas, VA, USA). 

All tested compounds were dissolved in DMSO (dimethyl sulfoxide, Sigma-Aldrich, Darmstadt, Germany) and then suspended in Minimum Essential Medium (MEM; Sigma-Aldrich, Darmstadt, Germany) supplemented with 2% heat-inactivated fetal bovine serum (FBS; Sigma-Aldrich, Darmstadt, Germany), 2 mM L-glutamine (Sigma-Aldrich, Darmstadt, Germany), and 100 units/mL penicillin G with 100 mg/mL streptomycin (Sigma-Aldrich, Darmstadt, Germany). 

Cells were propagated in Minimum Essential Medium (MEM; Sigma-Aldrich, Darmstadt, Germany) supplemented with 10% heat-inactivated fetal bovine serum (FBS; Sigma-Aldrich, Darmstadt, Germany) and 100 units/mL penicillin G with 100 mg/mL streptomycin (Sigma-Aldrich, Darmstadt, Germany). Upon reaching 80–90% confluency, cells were harvested with 0.25% trypsin in 1 mM EDTA (Life Technologies, Warsaw, Poland) and seeded into 96-well microplates at 2 × 10^5^ cells/mL. After overnight incubation at 37 °C in a humidified atmosphere containing 5% CO_2_, the culture medium was replaced with a 100 μL freshly prepared solution of tested compounds diluted with a maintenance medium supplemented with 2% FBS to obtain compound concentrations of 10 µM. The final concentration of DMSO in the medium was 0.1%. All experiments were carried out in triplicate. Compounds treated and untreated cells (control group) were incubated at 37 °C for 48 h in a humidified atmosphere containing 5% CO_2_. 

After incubation with drugs, the cells were treated with 3-(4,5-dimethylthiazol-2-yl)-2,5-diphenyltetrazolium bromide dye solution (MTT, Sigma-Aldrich, Darmstadt, Germany) (25 μL, 5 mg/mL) for 2 h and lysed with solvent solution (100 μL) containing: DMF (Sigma-Aldrich, Darmstadt, Germany) (45 mL), SDS (Sigma-Aldrich, Darmstadt, Germany) (13.5 g), and distilled water (55 mL). After overnight incubation at 37 °C, optical density at 550 nm and a reference wavelength of 670 nm were measured on a microplate spectrophotometer, Varioskan Lux (Thermo Fisher Scientific, Waltham, MA, USA). Compounds demonstrating cell viability ≥50% determined in both cytotoxicity as well as antiviral screening were selected for further studies.

#### 4.2.2. Cytotoxicity Assay in the Range of 0.1–1000 µM on Normal and Cancer Cell Lines

Cytotoxic properties of compounds **1e**–**1g, 1e[PF_6_]**, **8a**–**8d**, and **9a** were assessed on seven cell lines, including four normal cell lines: Vero (CCL-81, Cercopithecus aethiops normal kidney cells), LLC-MK2 (CCL-7, Macaca mulatta normal kidney cells), MRC-5 (CCL-171, Human lung normal fibroblasts), and NCTC clone 929 (CCL-1, Mus musculus normal subcutaneous connective tissue cells) and three cancer cell lines: A549 (CCL-185, Human lung carcinoma cells), HeLa (CCL-2, Human cervix adenocarcinoma cells), and HepG2 (HB-8065, Human hepatocellular carcinoma). Cell lines were purchased from the American Type Culture Collection (ATCC, Manassas, VA, USA).

All tested compounds were dissolved in DMSO (dimethyl sulfoxide, Sigma-Aldrich, Darmstadt, Germany) and then suspended in Minimum Essential Medium (MEM; Sigma-Aldrich, Darmstadt, Germany) supplemented with 2% heat-inactivated fetal bovine serum (FBS; Sigma-Aldrich, Darmstadt, Germany), 2 mM L-glutamine (Sigma-Aldrich, Darmstadt, Germany), and 100 units/mL penicillin G with 100 mg/mL streptomycin (Sigma-Aldrich, Darmstadt, Germany). 

Investigated cells were propagated in Minimum Essential Medium (MEM; Sigma-Aldrich, Darmstadt, Germany) supplemented with 10% heat-inactivated fetal bovine serum (FBS; Sigma-Aldrich, Darmstadt, Germany) and 100 units/mL penicillin G with 100 mg/mL streptomycin (Sigma-Aldrich, Darmstadt, Germany). After reaching 80–90% confluency, cells were harvested with trypsin (Life Technologies, Warsaw, Poland) and seeded into 96-well microplates at 2 × 10^5^ cells/mL. After overnight incubation at 37 °C in a humidified atmosphere containing 5% CO_2_, the culture medium was replaced with a 100 μL freshly prepared solution of tested compounds diluted with a maintenance medium supplemented with 2% FBS to obtain compound concentrations in the range from 0.1 to 1000 µM. The final concentration of DMSO in the medium was 0.1%. All experiments were carried out in triplicate. Cells exposed to investigated compounds and unexposed cells (control group) were incubated at 37 °C for 48 h in a humidified atmosphere containing 5% CO_2_ [37,38]. The cytotoxicity was evaluated by the MTT assay.

After the incubation, cells were treated for 2 h with 3-(4,5-dimethylthiazol-2-yl)-2,5-diphenyltetrazolium bromide dye solution (MTT, Sigma-Aldrich, Darmstadt, Germany) and lysed with solvent solution. After overnight incubation at 37 °C optical density at 550 nm, and a reference wavelength of 670 nm was measured on a microplate spectrophotometer, Varioskan Lux (Thermo Fisher Scientific, Waltham, MA, USA). The cytotoxic concentration (CC_50_) was defined as the concentration required to reduce cell viability by 50% compared to untreated controls and was calculated by linear regression analysis of the dose–response curves obtained from the data.

#### 4.2.3. Antiviral Screening of Compounds at a Concentration of 10 µM

Antiviral properties of compounds **1a**, **1c**, **1a[PF_6_]**, **1c[PF_6_]**, **9b[PF_6_]**, **9c[PF_6_]**, **10a**–**10d**, **2a**, and **2b** were assessed against against five viruses: Human herpesvirus 1 (HSV-1, VR-539), Human parainfluenza virus type 3 (HPIV-3, VR-93), Human adenovirus 5 (AdV5, VR-5), Human herpesvirus 5 (HCMV, VR-977), and Encephalomyocarditis virus (EMCV, VR-1479). Viruses were purchased from the American Type Culture Collection (ATCC, Manassas, VA, USA). 

All tested compounds were dissolved in DMSO (dimethyl sulfoxide, Sigma-Aldrich, Darmstadt, Germany) and then suspended in Minimum Essential Medium (MEM; Sigma-Aldrich, Darmstadt, Germany) supplemented with 2% heat-inactivated fetal bovine serum (FBS; Sigma-Aldrich, Darmstadt, Germany), 2 mM L-glutamine (Sigma-Aldrich, Darmstadt, Germany), and 100 units/mL penicillin G with 100 mg/mL streptomycin (Sigma-Aldrich, Darmstadt, Germany). 

Vero, LLC-MK2, NCTC clone 929, MRC-5, and HeLa cells were propagated in Minimum Essential Medium (MEM; Sigma-Aldrich, Darmstadt, Germany) supplemented with 10% heat-inactivated fetal bovine serum (FBS; Sigma-Aldrich, Darmstadt, Germany) and 100 units/mL penicillin G with 100 mg/mL streptomycin (Sigma-Aldrich, Darmstadt, Germany). Upon reaching 80–90% confluency, cells were harvested with 0.25% trypsin in 1 mM EDTA (Life Technologies, Warsaw, Poland) and seeded onto 96-well microplates at 2 × 10^5^ cells/mL. After overnight incubation of cells at 37 °C in a humidified atmosphere containing 5% CO_2_, the culture medium was removed, and cells were inoculated with the respective virus solution in MEM supplemented with 2% FBS and antibiotics (HSV-1 MOI 0.005, 1000 virions/mL; HPIV-3 MOI 0.01, 2000 virions/mL; AdV5 MOI 0.005, 1000 virions/mL, EMCV MOI 0.005, 1000 virions/mL, HCMV 20 PFU (plaque forming units) per well). After a 1-h (HSV-1, HPIV-3, AdV5, and EMCV) or 2-h adsorption period (HCMV), the residual virus was removed, and the infected cells were further incubated with a 100 μL freshly prepared solution of tested compounds diluted with a maintenance medium supplemented with 2% FBS to obtain compound concentrations of 10 µM. The final concentration of DMSO in the medium was 0.1%. All experiments were carried out in triplicate. The cells monolayers were incubated with the compounds at 37 °C in a humidified atmosphere containing 5% CO_2_ until the typical cytopathic effect (CPE) was visible. Viral infection was evaluated by the MTT assay or plaque reduction assay (HCMV). After incubation with drugs, the cells were treated with 3-(4,5-dimethylthiazol-2-yl)-2,5-diphenyltetrazolium bromide dye solution (MTT, Sigma-Aldrich, Darmstadt, Germany) (25 μL, 5 mg/mL) for 2 h and lysed with solvent solution (100 μL) containing: DMF (Sigma-Aldrich, Darmstadt, Germany) (45 mL), SDS (Sigma-Aldrich, Darmstadt, Germany) (13.5 g), and distilled water (55 mL). After overnight incubation at 37 °C, the optical density at 550 nm and a reference wavelength of 670 nm were measured on a microplate spectrophotometer, Varioskan Lux (Thermo Fisher Scientific, Waltham, MA, USA). The number of HCMV plaques was counted under an inverted microscope Olympus IX73 (Olympus, Tokyo, Japan). Compounds demonstrating cell viability ≥50% determined with both cytotoxicity as well as antiviral screening, were selected for further studies.

#### 4.2.4. Antiviral Activity Assay in the Range of 0.1–1000 µM

Antiviral properties of compounds **1e**–**1g, 1e[PF_6_]**, **8a**–**8d**, and **9a** were assessed against five viruses: Human herpesvirus 1 (HSV-1, VR-539), Human parainfluenza virus type 3 (HPIV-3, VR-93), Human adenovirus 5 (AdV5, VR-5), Human herpesvirus 5 (HCMV, VR-977), and Encephalomyocarditis virus (EMCV, VR-1479). Viruses were purchased from the American Type Culture Collection (ATCC, Manassas, VA, USA).

All tested compounds were dissolved in DMSO (dimethyl sulfoxide, Sigma-Aldrich, Darmstadt, Germany) and then suspended in Minimum Essential Medium (MEM; Sigma-Aldrich, Darmstadt, Germany) supplemented with 2% heat-inactivated fetal bovine serum (FBS; Sigma-Aldrich, Darmstadt, Germany), 2 mM L-glutamine (Sigma-Aldrich, Darmstadt, Germany), and 100 units/mL penicillin G with 100 mg/mL streptomycin (Sigma-Aldrich, Darmstadt, Germany).

Investigated cells were propagated in Minimum Essential Medium (MEM; Sigma-Aldrich, Darmstadt, Germany) supplemented with 10% heat-inactivated fetal bovine serum (FBS; Sigma-Aldrich, Darmstadt, Germany) and 100 units/mL penicillin G with 100 mg/mL streptomycin (Sigma-Aldrich, Darmstadt, Germany). After reaching 80–90% confluency, cells were harvested with trypsin (Life Technologies, Warsaw, Poland) and seeded into 96-well microplates at 2 × 10^5^ cells/mL after overnight incubation of cells at 37 °C in a humidified atmosphere containing 5% CO_2_.

The culture medium was removed from confluent cells grown in 96-well microplates, and the cells were inoculated with virus solutions in MEM supplemented with 2% FBS and antibiotics (HSV-1 MOI 0.005, 1000 virions/mL; HPIV-3 MOI 0.01, 2000 virions/mL; AdV5 MOI 0.005, 1000 virions/mL; EMCV 0.005, 1000 virions/mL; HCMV 20 PFU/well (plaque-forming units per well). After a 1-h (HSV-1, HPIV-3, AdV5, and EMCV) or 2-h adsorption period (HCMV), residual viral particles were removed, and infected cells were further incubated with MEM supplemented with 2% FBS containing compound concentrations in the range from 0.1 to 1000 µM [38]. All experiments were carried out in triplicate. The cell monolayers were incubated with the investigated compounds at 37 °C in a humidified atmosphere containing 5% CO_2_ until a cytopathic effect (CPE) was visible. Viral infection was evaluated by the MTT assay (as described previously) or plaque reduction assay. HCMV plaques were counted under an inverted microscope, Olympus IX73 (Olympus, Tokyo, Japan). Antiviral activity was expressed as the concentration required to reduce the number of viral plaques to 50% of the control (virus-infected but untreated).

## Figures and Tables

**Table 1:** Cytotoxic effect of compounds **1e**–**1g, 1e[PF_6_]**, **8a**–**8d**, and **9a** on the normal and cancer cell lines.

Compound	CC_50_ [µM]						
	Normal Cell Lines				Cancer Cell Lines		
	Vero	LLC-MK2	NCTC Clone 929	MRC-5	HeLa	A549	HepG2
**1e**	22.667 ± 1.528	53.500 ± 2.179	79.167 ± 1.443	52.667 ± 0.577	0.039 ± 0.001	37.667 ± 3.786	423.333 ± 7.638
**1e[PF_6_]**	56.333 ± 3.215	150.333 ± 2.517	148.333 ± 11.547	149.667 ± 4.167	0.080 ± 0.001	38.000 ± 3.464	316.667 ± 5.774
**1f**	38.000 ± 4.000	59.000 ± 1.000	48.333 ± 2.887	36.667 ± 2.309	0.039 ± 0.001	1.600 ± 0.173	152.000 ± 2.000
**1g**	0.107 ± 0.012	66.167 ± 2.255	30.000 ± 2.000	54.333 ± 1.155	0.053 ± 0.006	10.333 ± 1.041	130.000 ± 5.000
**8a**	250.000 ± 10.000	326.677 ± 7.640	256.611 ± 10.410	206.170 ± 8.611	20.000 ± 3.460	7.500 ± 0.500	361.667 ± 2.890
**8b**	143.333 ± 2.890	256.667 ± 6.770	90.667 ± 4.160	24.333 ± 2.080	7.333 ± 0.760	5.500 ± 0.500	162.667 ± 6.430
**8c**	53.667 ± 1.533	55.000 ± 1.000	28.500 ± 0.500	45.667 ± 3.790	16.000 ± 1.000	7.370 ± 0.320	95.000 ± 1.000
**8d**	229.333 ± 9.020	7.500 ± 1.000	283.333 ± 10.410	59.667 ± 2.520	5.500 ± 0.500	5.070 ± 0.120	321.670 ± 5.770
**9a**	0.347 ± 0.006	0.040 ± 0.002	3.750 ± 0.087	0.090 ± 0.010	0.019 ± 0.001	0.035 ± 0.005	6.900 ± 0.361

**Table 2:** Antiviral activity of compounds **1e**–**1g**, **1e[PF_6_]**, **8a**–**8d**, and **9a**.

Compound	IC_50_ [µM]				
	HSV-1	HPIV-3	EMCV	HCMV	AdV5
**1e**	>22.667	>53.500	>79.167	>52.667	>0.039
**1e[PF_6_]**	>56.333	>150.333	>148.333	>149.667	>0.080
**1f**	>38.000	>59.000	>48.333	>36.667	>0.039
**1g**	>0.107	>66.167	>30.000	>54.333	>0.053
**8a**	>250.000	>326.667	>256.611	>206.170	>20.000
**8b**	>143.333	>256.667	>90.667	>24.333	>7.333
**8c**	>53.667	>55.000	>28.500	>45.667	>16.000
**8d**	>229.333	>7.500	>283.333	>59.667	>5.500
**9a**	>0.347	>0.040	>3.750	>0.090	>0.019

## Data Availability

All data are available in this publication.

## Supplementary Materials

The following supporting information can be downloaded at https://www.mdpi.com/article/10.3390/molecules27113524/s1: Synthetic protocols for the preparation of imidazole *N*-oxides **6**, imidazoles **7**, and copies of ^1^H and ^13^C NMR spectra of all the new imidazole *N*-oxides **6** and imidazolium salts of types **1**, **8**, and **9**. Results of the initial cytotoxicity and antiviral screening of the selected lepidilines and their known analogues, i.e., **1a**, **1c**, **1a[PF_6_]**, **1c[PF_6_]**, **2a**, **2b**, **9b[PF_6_]**, **9c[PF_6_]**, and **10a**–**10d**).
