# Supplementary material for: Fluorinated Analogues of Lepidilines A and C: Synthesis and Screening of Their Anticancer and Antiviral Activity
Source: Molecules. 2022 May 30;27(11):3524. doi: 10.3390/molecules27113524 (PMC9181938; doi:10.3390/molecules27113524)
Supplement: Supplementary file 1 [file molecules-27-03524-s001.zip › molecules-1742052-supplementary.pdf]

Supporting Information

for

Fluorinated analogues of lepidilines A and C: Synthesis and screening of their anticancer and antiviral activity

Grzegorz Mlostoń[a,*], Mateusz Kowalczyk[a], Małgorzata Celeda[a], Marcin Jasiński[a,*], Marta Denel-Bobrowska[b] and Agnieszka B. Olejniczak[b,*]

[a] *Faculty of Chemistry, University of Lodz, Tamka 12, 91403 Łódź, Poland*

[b] *Institute of Medical Biology, Polish Academy of Sciences, 106 Lodowa St., 93232 Łódź, Poland*

Content

1. Synthetic details S2

2. Copies of ^{1}H and ^{13}C NMR spectra S4

3. Supplementary biological results S22

4. References S24

1. Synthetic details

Synthesis and complete spectroscopic characterization of lepidilines A and C (**1a**, **1c**), imidazolium hexafluorophosphates **1a[PF$_6$]**, **9b[PF$_6$]**, **9c[PF$_6$]** and imidazole-2-thiones **10a**-**10d** have been reported elsewhere [1].

1.1. Synthesis of imidazole *N*-oxides **6a**-**6f**.

Preparation of formaldimines **3**: A mixture of the corresponding benzylamine **4** (2.5 mmol) and aqueous formaldehyde (37%, 7.5 mmol) in benzene (10 mL) was refluxed in a Dean−Stark apparatus for 2 h. The solvent was removed under reduced pressure to give crude product **3** (quant.) as thick oil, which was used for the next step without purification.

Preparation of imidazole *N*-oxides **6**: A solution of α-hydroxyiminoketone **5** (2.0 mmol) and crude formaldimine **3** (2.5 mmol) in glacial acetic acid (6 mL) was stirred at room temperature overnight. Then, excess concentrated HCl was added dropwise (1.0 mL) the solvents were removed under reduced pressure. The resulting material was dissolved in MeOH (10 mL), excess solid $NaHCO_3$ (2.0 g) was added the stirring was continued for 30 min until the evolution of CO_2 ceased. The solvent was removed in vacuo, CH_2Cl_2 (15 mL) was added and the precipitates were separated by filtration through Celite®. After solvent was removed, the residue was triturated with Et_2O (10 mL), the precipitate identified as spectroscopically pure imidazole *N*-oxide **6** was separated and used for the next step without further purification.

1-(3-Fluorobenzyl)-4,5-dimethylimidazole 3-oxide (**6a**): 291 mg (66%), beige solid, m.p. 176–178 °C. ^{1}H NMR (600 MHz, $CDCl_3$): δ 2.07, 2.21 (2 s, 3 H each, 2 Me), 4.98 (s, 2 H, CH_2), 6.77-6.79, 6.84-6.86, 7.02-7.05, 7.32-7.36 (4 m, 1 H each), 7.85 (s, 1 H, C(2)H). ^{13}C NMR (151 MHz, $CDCl_3$): δ 7.5, 8.9, 48.9 (d, $^4J_{C\text{-}F}$ = 1.6 Hz, CH_2), 113.9 (d, $^2J_{C\text{-}F}$ = 22.5 Hz, CH), 115.8 (d, $^2J_{C\text{-}F}$ = 21.1 Hz, CH), 121.4, 122.3 (d, $^4J_{C\text{-}F}$ = 3.2 Hz, CH), 124.8, 127.8, 131.1 (d, $^3J_{C\text{-}F}$ = 8.3 Hz, CH), 137.2 (d, $^3J_{C\text{-}F}$ = 7.0 Hz, *i*-C), 163.3 (d, $^1J_{C\text{-}F}$ = 248.3 Hz, *i*-C). ^{19}F NMR (565 MHz, $CDCl_3$): δ –111.2 (m$_c$, CF). IR (neat): *v* 1386, 1334, 1252, 1133, 991, 917 cm^{-1}. ESI-MS (*m*/*z*): 221.3 (100, [*M*+H]$^+$).

4,5-Dimethyl-1-[3-(trifluoromethoxy)benzyl]imidazole 3-oxide (**6b**): 492 mg (86%), yellow solid, m.p. 158–160 °C. ^{1}H NMR (600 MHz, $CDCl_3$): δ 2.05, 2.18 (2 s, 3 H each, 2 Me), 4.99 (s, 2 H, CH_2), 6.93 (m$_c$, 1 H), 6.96-6.98, 7.16-7.19, 7.37-7.40 (3 m, 1 H each), 7.82 (s, 1 H, C(2)H). ^{13}C NMR (151 MHz, $CDCl_3$): δ 7.5, 8.9, 48.8, 119.4, 120.4 (q, $^1J_{C\text{-}F}$ = 257.9 Hz, OCF_3), 121.0, 121.3, 124.6, 124.9, 127.9, 130.9, 137.2, 149.9. ^{19}F NMR (565 MHz, $CDCl_3$): δ –57.9 (s, OCF_3). IR (neat): *v* 1390, 1334, 1249, 1208, 1163, 1084, 984 cm^{-1}. ESI-MS (*m*/*z*): 309.3 (20, [*M*+Na]$^+$), 287.4 (100, [*M*+H]$^+$).

4,5-Dimethyl-1-[3-(trifluoromethyl)benzyl]imidazole 3-oxide (**6c**): 393 mg (73%), colorless solid, m.p. 250–253 °C (decomp.). ^{1}H NMR (600 MHz, $CDCl_3$): δ 2.07, 2.21 (2 s, 3 H each, 2 Me), 5.04 (s, 2 H, CH_2), 7.21-7.23 (m, 1 H), 7.38 (m$_c$, 1 H), 7.48-7.52, 7.59-7.62 (2 m, 1 H each), 7.87 (s, 1 H, C(2)H). ^{13}C NMR (151 MHz, $CDCl_3$): δ 7.5, 9.0, 49.0, 121.3, 123.6 (q, $^3J_{C\text{-}F}$ = 3.7 Hz, CH), 123.7 (q, $^1J_{C\text{-}F}$ = 272.6 Hz, CF_3), 124.8, 125.7 (q, $^3J_{C\text{-}F}$ = 3.7 Hz, CH), 128.1, 130.0(br), 130.1, 131.9 (q, $^2J_{C\text{-}F}$ = 32.9 Hz, *i*-C), 135.8. ^{19}F NMR (565 MHz, $CDCl_3$): δ –62.7 (s, CF_3). IR (neat): *v* 1327, 1166, 1182, 1111, 1074, 972 cm^{-1}. ESI-MS (*m*/*z*): 271.3 (100, [*M*+H]$^+$).

4,5-Dimethyl-1-[2-(trifluoromethyl)benzyl]imidazole 3-oxide (**6d**): 270 mg (50%), beige solid, m.p. 142–144 °C. ^{1}H NMR (600 MHz, $CDCl_3$): δ 2.02, 2.23 (2 s, 3 H each, 2 Me), 5.19 (s, 2 H, CH_2), 6.78-6.80, 7.44-7.47, 7.50-7.54, 7.71-7.74 (4 m, 1 H each), 7.81 (s, 1 H, C(2)H). ^{13}C NMR (151 MHz, $CDCl_3$): δ 7.5, 8.6, 45.8* (q, $J_{C\text{-}F}$ = 3.4 Hz, CH_2), 121.5, 124.1 (q, $^1J_{C\text{-}F}$ = 273.8 Hz, CF_3), 124.9(br), 126.6 (q, $^3J_{C\text{-}F}$ = 5.6 Hz, CH), 127.4, 127.5 (q, $^2J_{C\text{-}F}$ = 31.0 Hz, *i*-C), 127.8, 128.7, 133.0(br), 133.4(br); *through-space C–F coupling. ^{19}F NMR (565 MHz, $CDCl_3$): δ –60.5 (s, CF_3). IR (neat): *v* 1312, 1163, 1103, 1036, 775 cm^{-1}. ESI-MS (*m*/*z*): 271.4 (100, [*M*+H]$^+$).

4,5-Dimethyl-1-[4-(trifluoromethyl)benzyl]imidazole 3-oxide (**6e**): 411 mg (76%), beige solid, m.p. 153–154 °C. ^{1}H NMR (600 MHz, $CDCl_3$): δ 2.00, 2.14 (2 s, 3 H each, 2 Me), 5.06 (s, 2 H, CH_2), 7.15-7.18, 7.56-7.59 (2 m, 2 H each), 8.04 (s, 1 H, C(2)H). ^{13}C NMR (151 MHz, $CDCl_3$): δ 7.4, 8.8, 48.9, 121.4, 123.8 (q, $^1J_{C\text{-}F}$ = 272.4 Hz, CF_3), 125.1, 126.3 (q, $^3J_{C\text{-}F}$ = 3.5 Hz, 2 CH), 127.0, 127.7, 130.9 (q, $^2J_{C\text{-}F}$ = 32.8 Hz, *i*-C), 138.8 (br). ^{19}F NMR (565 MHz, $CDCl_3$): δ –62.8 (s, CF_3). IR (neat): ν 1320, 1163, 1111, 1066, 1018, 831 cm^{-1}. ESI-MS (*m/z*): 293.3 (18, [*M*+Na]$^+$), 271.4 (100, [*M*+H]$^+$).

4,5-Diphenyl-1-[3-(trifluoromethyl)benzyl]imidazole 3-oxide (**6f**): 355 mg (45%), colorless solid, m.p. 166–168 °C. ^{1}H NMR (600 MHz, $CDCl_3$): δ 5.02 (s, 2 H, CH_2), 7.15-7.19, 7.23-7.29, 7.36-7.39, 7.41-7.48, 7.55-7.59 (5 m, 3 H, 4 H, 2 H, 2 H, 3 H), 8.07 (s, 1 H, C(2)H). ^{13}C NMR (151 MHz, $CDCl_3$): δ 49.5, 123.7 (q, $^1J_{C\text{-}F}$ = 272.6 Hz, CF_3), 124.4 (q, $^3J_{C\text{-}F}$ = 3.6 Hz, CH), 125.8 (q, $^3J_{C\text{-}F}$ = 3.7 Hz, CH), 125.9, 126.8, 127.4, 127.5, 128.3, 128.5(br), 129.4, 129.7, 129.96, 130.01, 130.8(br), 130.9, 131.4, 131.7 (q, $^2J_{C\text{-}F}$ = 32.7 Hz, *i*-C), 135.8. ^{19}F NMR (565 MHz, $CDCl_3$): δ –62.8 (s, CF_3). IR (neat): ν 1450, 1323, 1204, 1163, 1126, 1074 cm^{-1}. ESI-MS (*m/z*): 394.3 (100, [*M*+H]$^+$).

1.2. Synthesis of imidazoles **7a**-**7d**.

A solution of imidazole *N*-oxide **6** (1.0 mmol) in MeOH (5.0 mL) was treated with an excess of freshly prepared Raney-nickel in MeOH. The mixture was stirred at room temperature until the starting *N*-oxide was fully consumed (monitored by TLC; typically ca. 1 h). The mixture was filtered and the solvent was removed under reduced pressure to give spectroscopically pure imidazole **7** which was used for the next step without further purification.

1-(3-Fluorobenzyl)-4,5-dimethylimidazole (**7a**): 157 mg (77%), brown solid, m.p. 59–62 °C. ^{1}H NMR (600 MHz, $CDCl_3$): δ 1.98, 2.15 (2 s, 3 H each, 2 Me), 4.99 (s, 2 H, CH_2), 6.68-6.71, 6.79-6.81, 6.94-6.97, 7.27-7.30 (4 m, 1 H each), 7.40 (s, 1 H, C(2)H). ^{13}C NMR (151 MHz, $CDCl_3$): δ 8.5, 12.9, 48.2(br), 113.6 (d, $^2J_{C,F}$ = 22.2 Hz, CH), 114.9 (d, $^2J_{C,F}$ = 21.0 Hz, CH), 122.1 (d, $^4J_{C,F}$ = 2.7 Hz, CH), 122.5, 130.6 (d, $^3J_{C,F}$ = 8.3 Hz, CH), 134.8, 135.5, 139.4 (d, $^3J_{C,F}$ = 7.1 Hz, *i*-C), 163.3 (d, $^1J_{C\text{-}F}$ = 247.4 Hz, *i*-C).

4,5-Dimethyl-1-[3-(trifluoromethoxy)benzyl]imidazole (**7b**): 235 mg (87%), colorless oil. ^{1}H NMR (600 MHz, $CDCl_3$): δ 1.98, 2.15 (2 s, 3 H each, 2 Me), 5.01 (s, 2 H, CH_2), 6.83-6.97 (m, 2 H), 7.13 (m_c, 1 H), 7.31-7.36 (m, 1 H) 7.40 (s, 1 H, C(2)H).

4,5-Dimethyl-1-[3-(trifluoromethyl)benzyl]imidazole (**7c**): 168 mg (66%), colorless solid, m.p. 80–83 °C. ^{1}H NMR (600 MHz, $CDCl_3$): δ 1.98, 2.16 (2 s, 3 H each, 2 Me), 5.05 (s, 2 H, CH_2), 7.14-7.16 (m 1 H), 7.33 (m_c, 1 H), 7.41 (s, 1 H, C(2)H), 7.42-7.46, 7.53-7.55 (2 m, 1 H each).

4,5-Diphenyl-1-[3-(trifluoromethyl)benzyl]imidazole (**7d**): 257 mg (68%), colorless solid, 101–103 °C. ^{1}H NMR (600 MHz, $CDCl_3$): δ 5.03 (s, 2 H, CH_2), 7.12-7.22, 7.36-7.42, 7.49-7.52 (3 m, 7 H, 4 H, 3 H), 7.69 (s, 1 H, C(2)H).

2. Copies of ^{1}H and ^{13}C NMR spectra

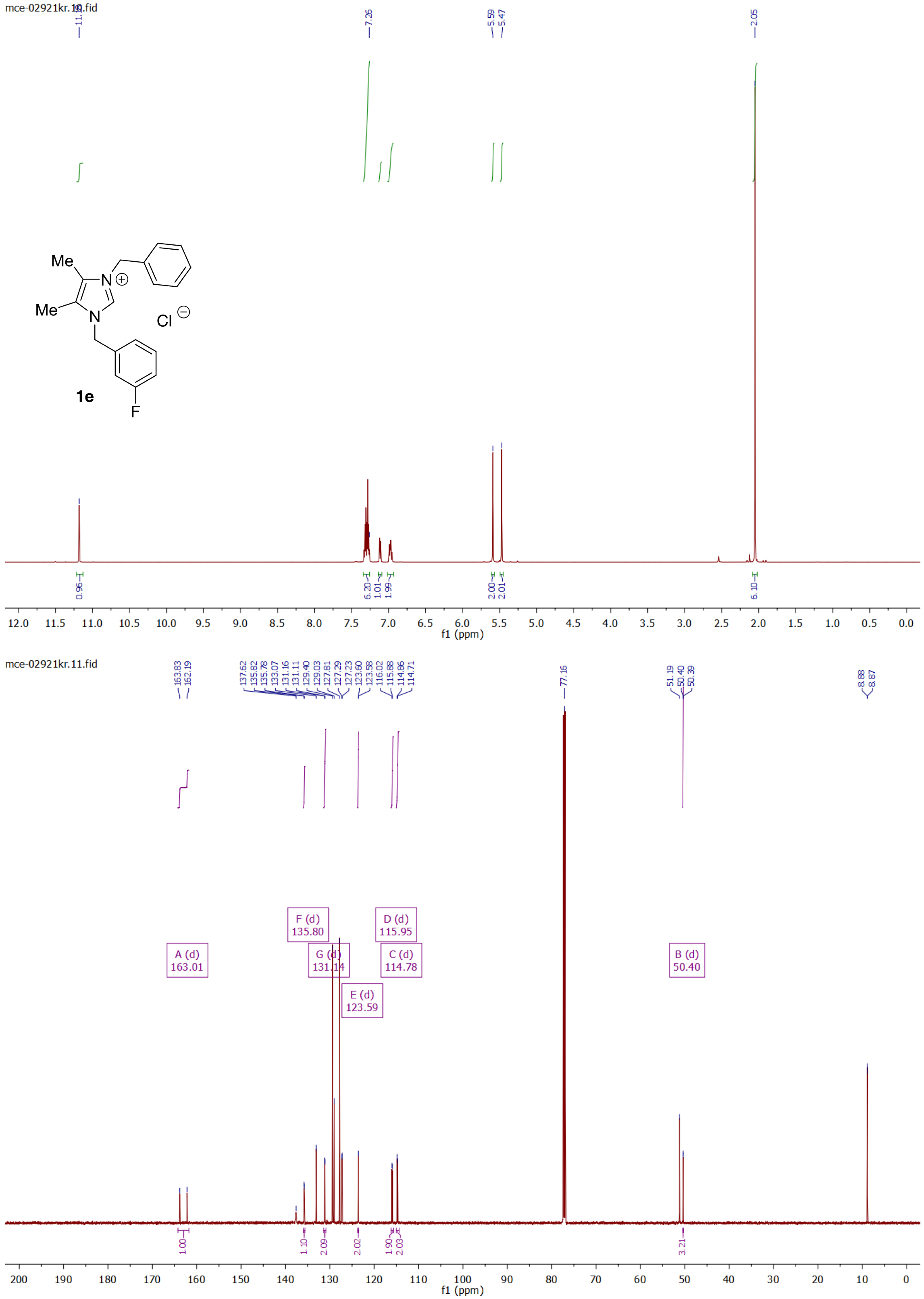

Figure S1. ^{1}H NMR (600 MHz, $CDCl_3$) and ^{13}C NMR (151 MHz, $CDCl_3$) spectra for compound **1e**.

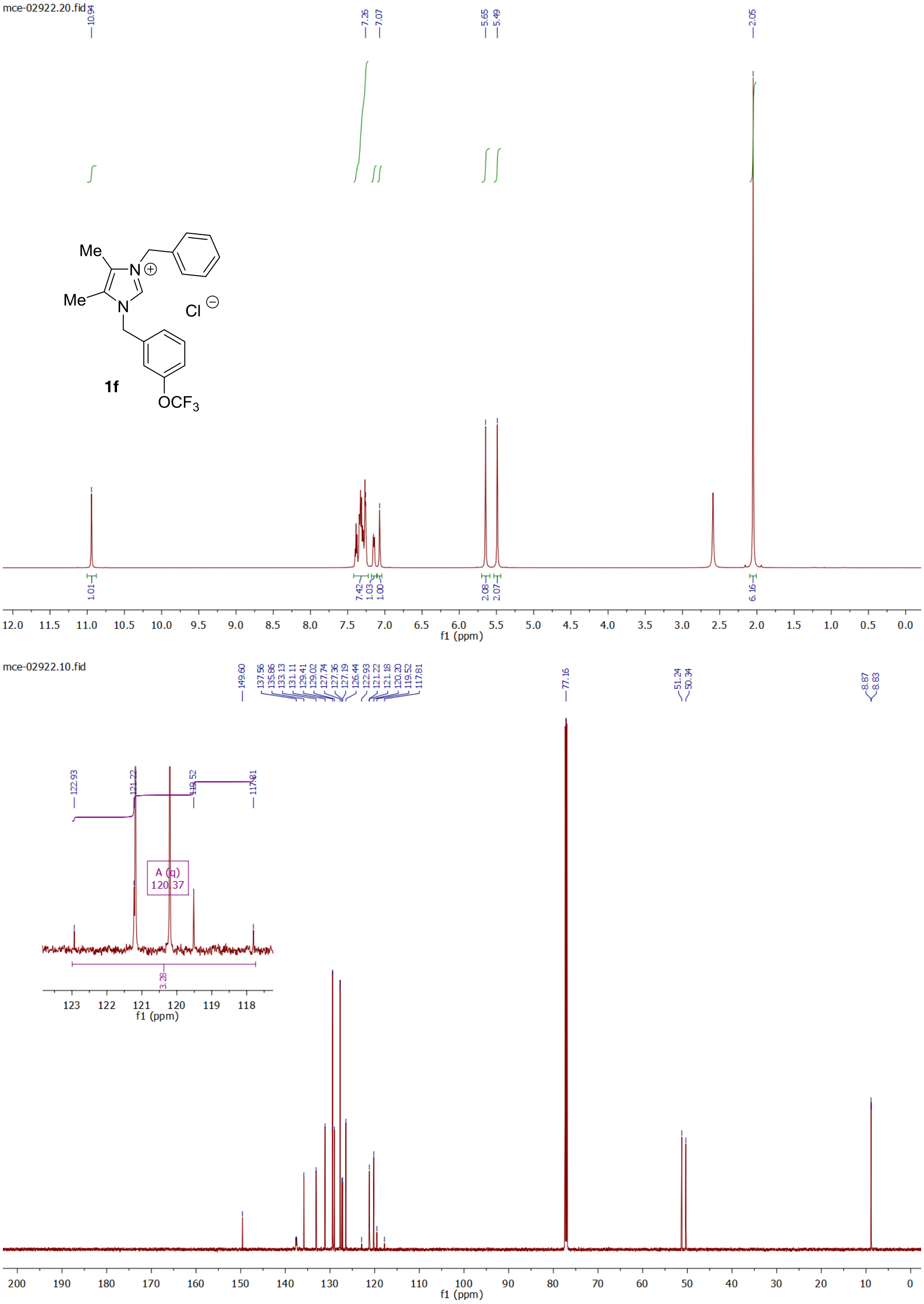

Figure S2. ^{1}H NMR (600 MHz, $CDCl_3$) and ^{13}C NMR (151 MHz, $CDCl_3$) spectra for compound **1f**.

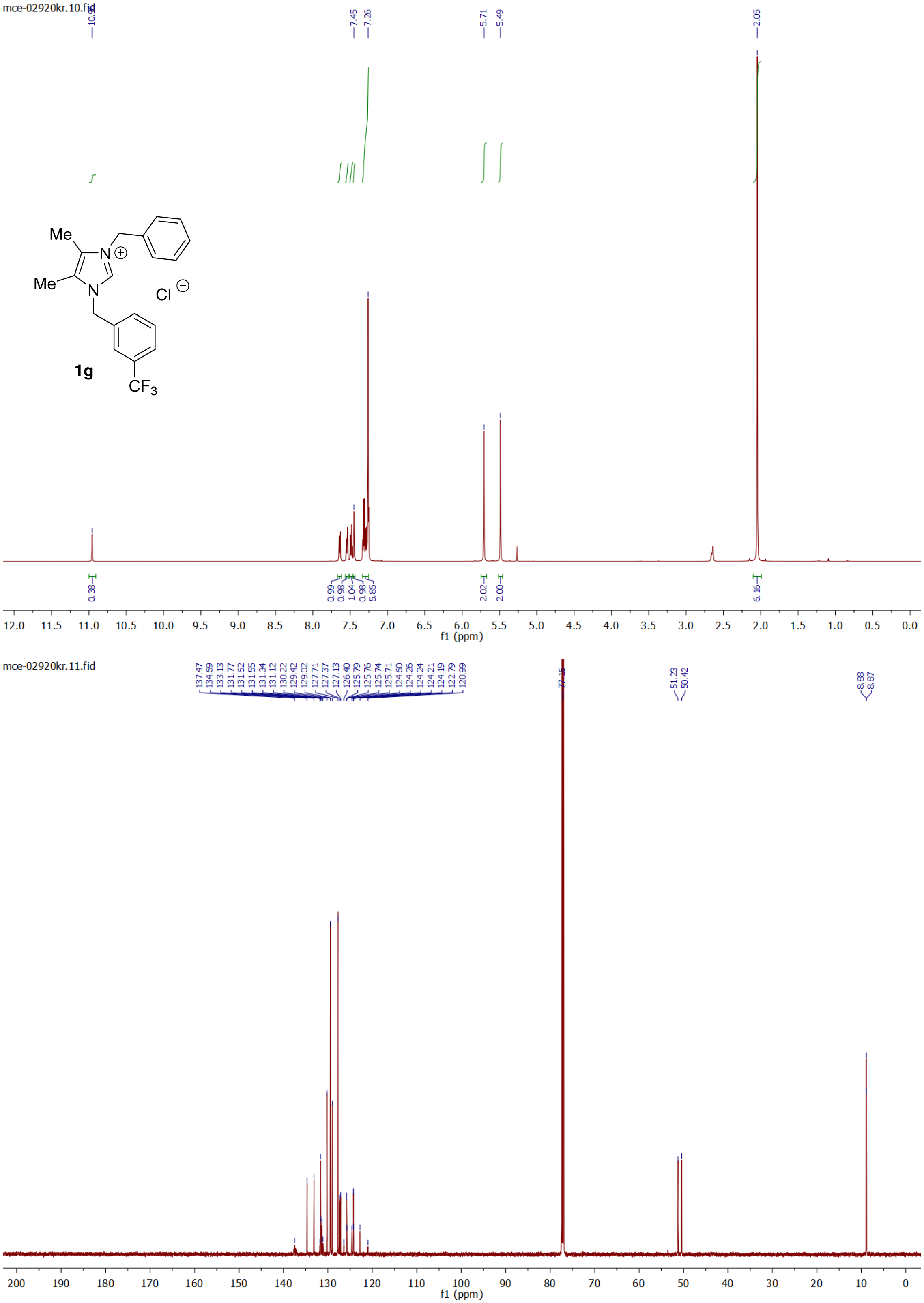

Figure S3. ^{1}H NMR (600 MHz, $CDCl_3$) and ^{13}C NMR (151 MHz, $CDCl_3$) spectra for compound **1g**.

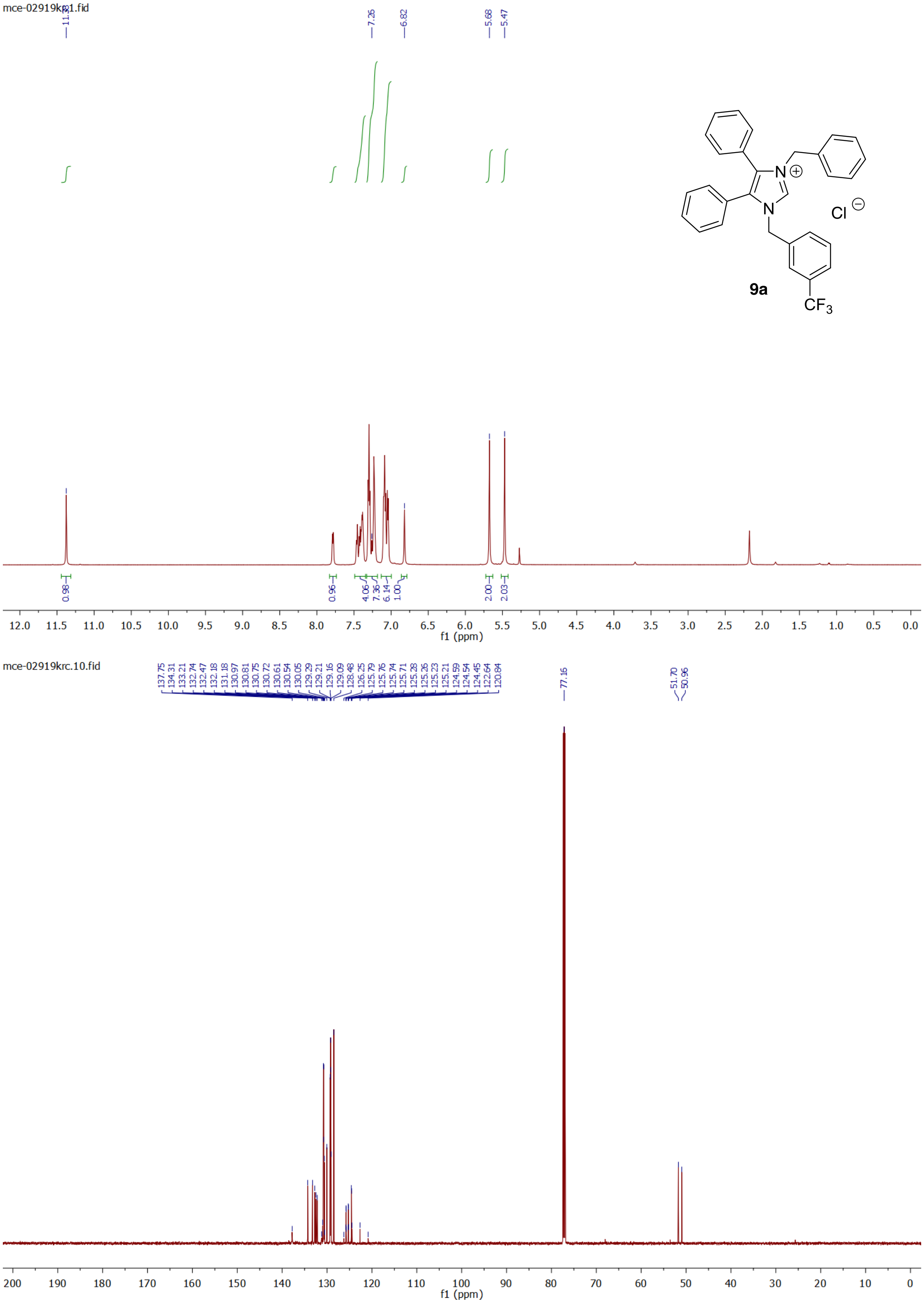

Figure S4. ^{1}H NMR (600 MHz, $CDCl_3$) and ^{13}C NMR (151 MHz, $CDCl_3$) spectra for compound **9a**.

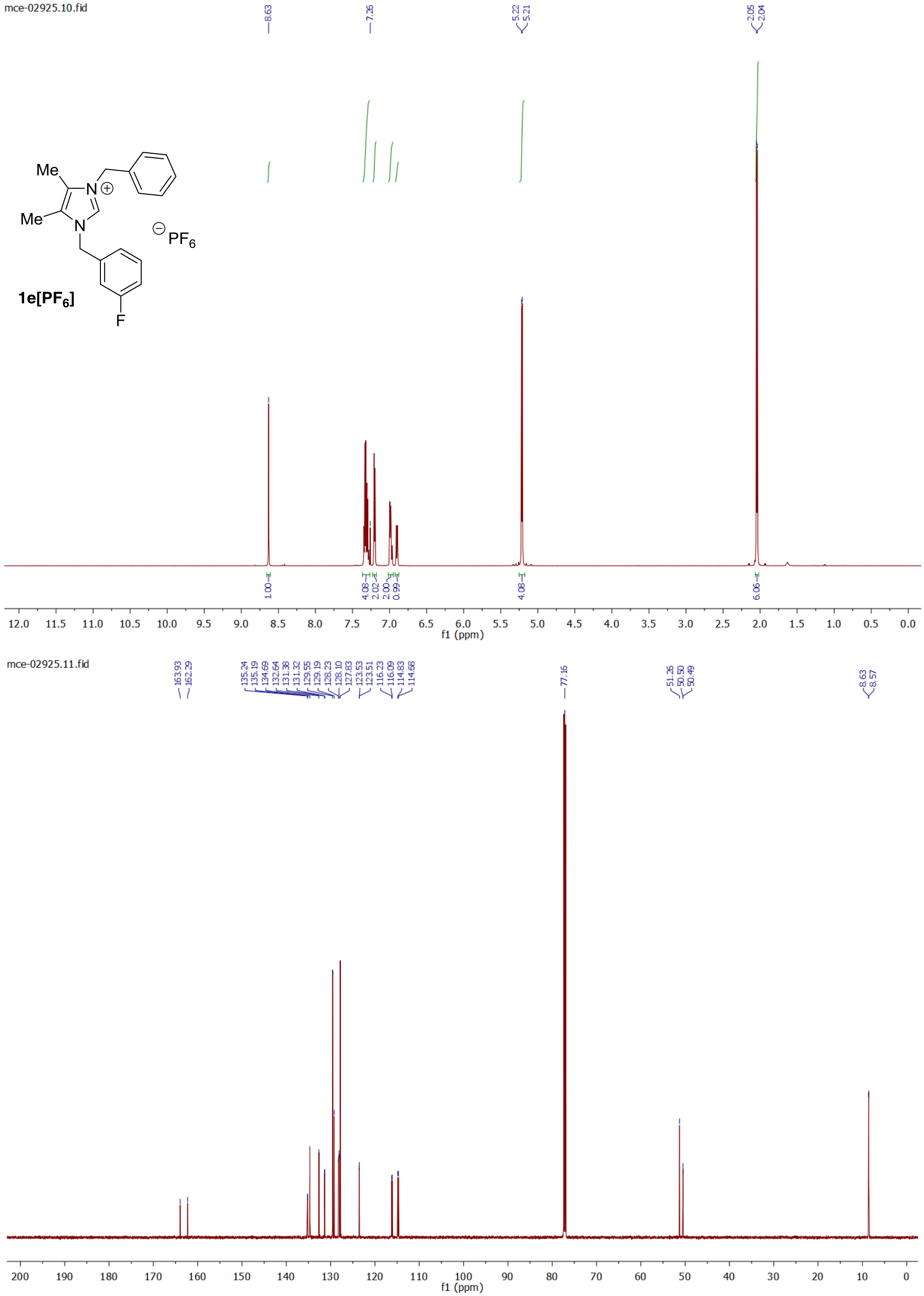

Figure S5. ^{1}H NMR (600 MHz, $CDCl_3$) and ^{13}C NMR (151 MHz, $CDCl_3$) spectra for compound **1e[PF6]**.

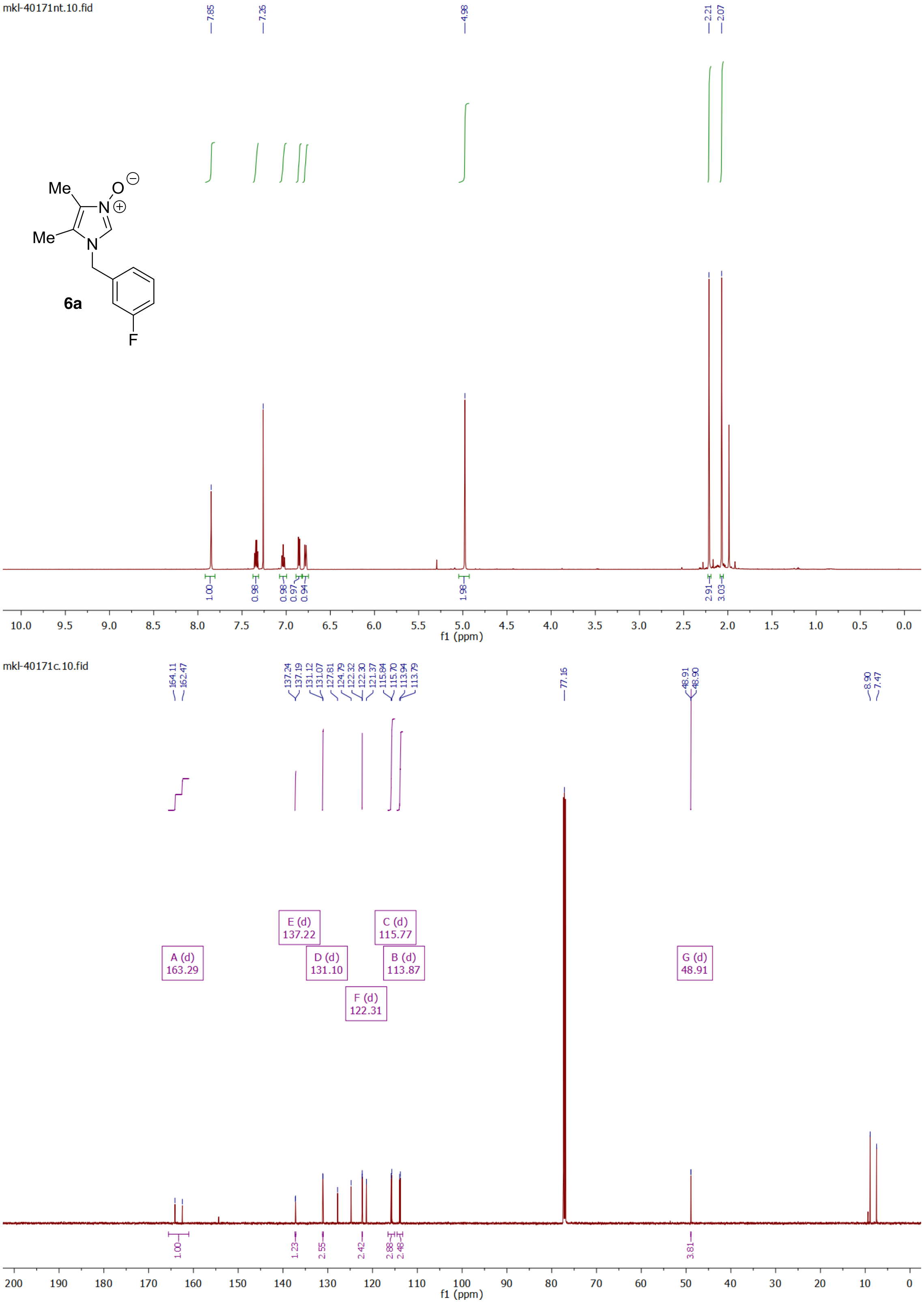

Figure S6. ^{1}H NMR (600 MHz, $CDCl_3$) and ^{13}C NMR (151 MHz, $CDCl_3$) spectra for compound **6a**.

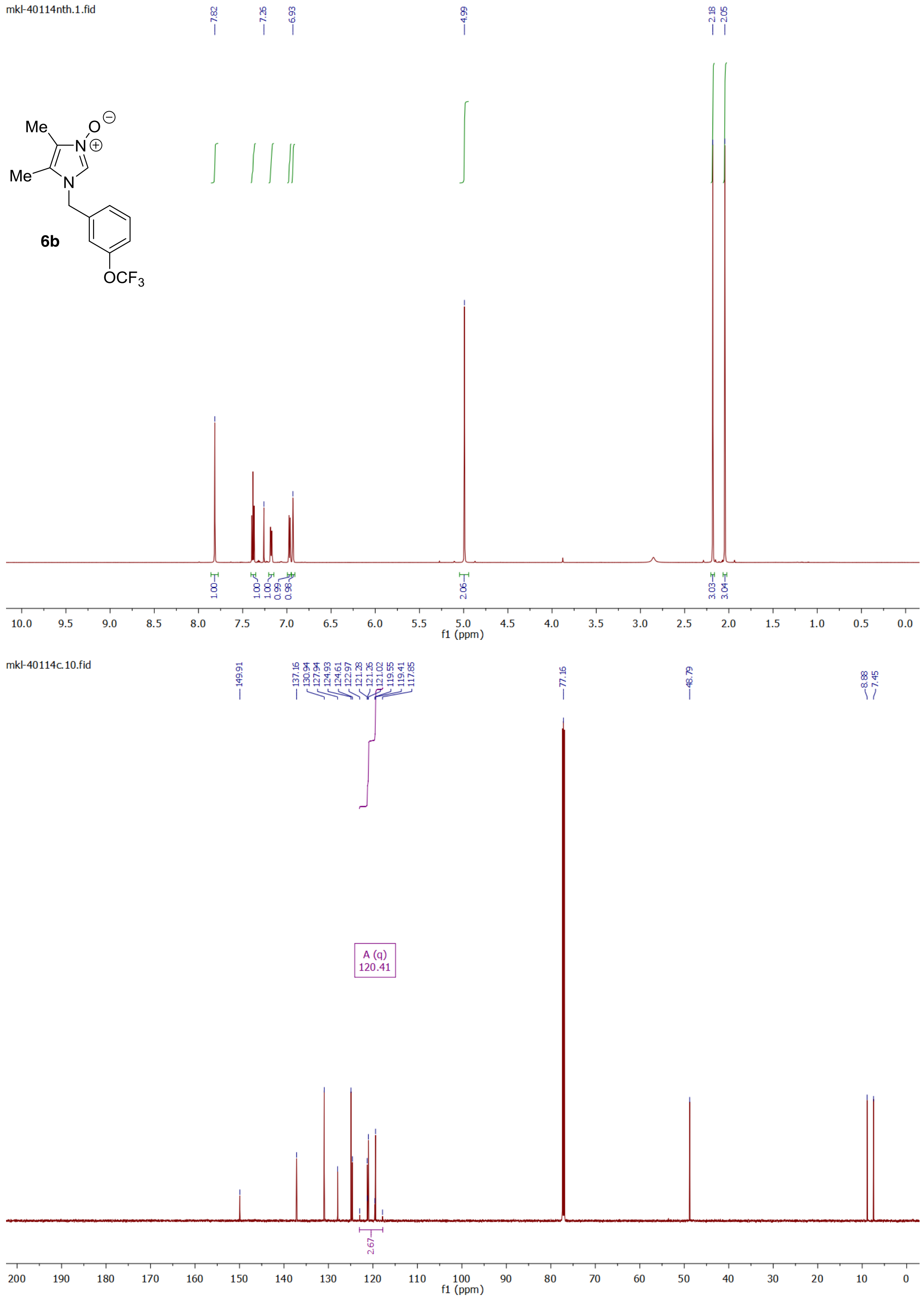

Figure S7. ^{1}H NMR (600 MHz, $CDCl_3$) and ^{13}C NMR (151 MHz, $CDCl_3$) spectra for compound **6b**.

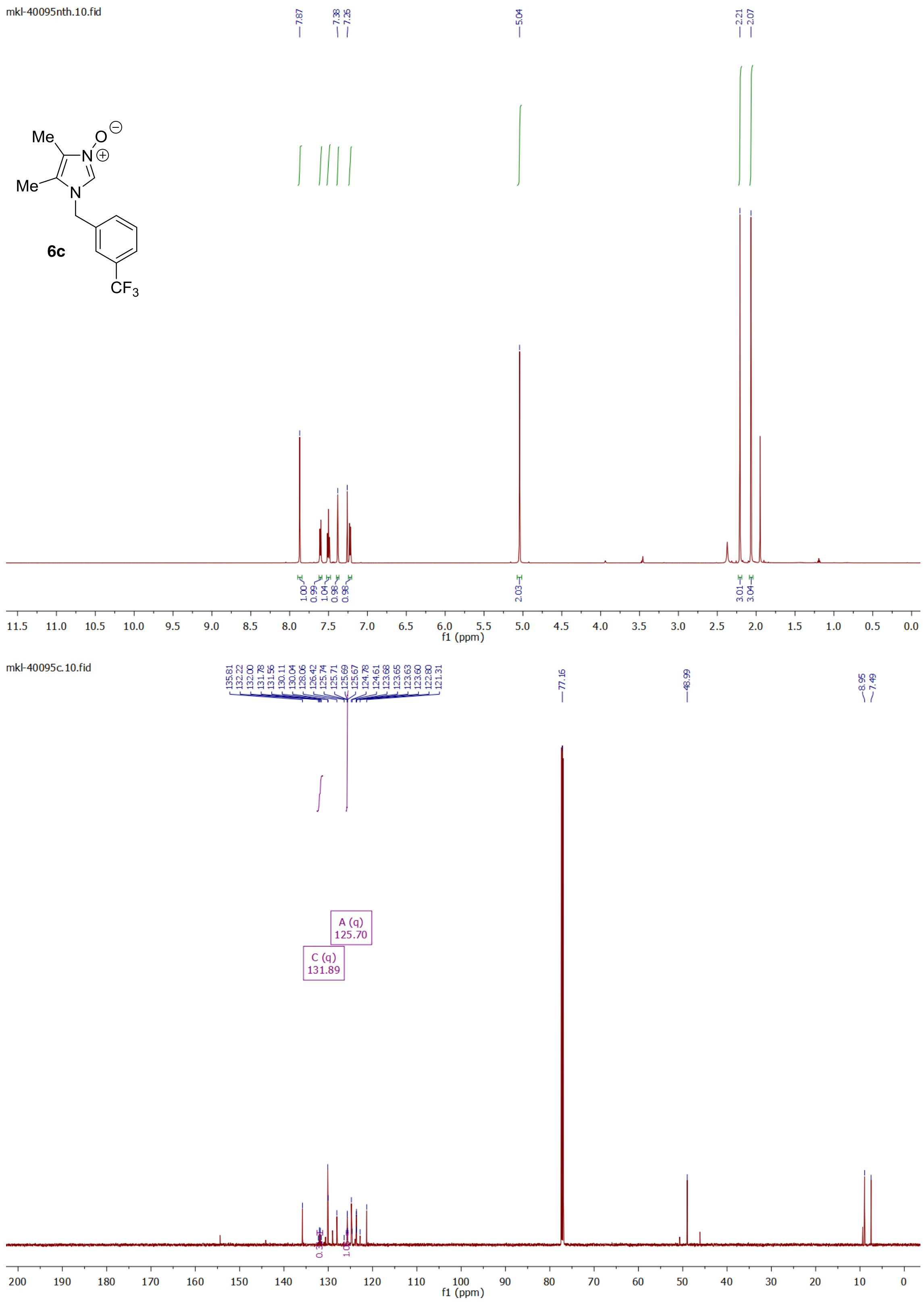

Figure S8. ^{1}H NMR (600 MHz, $CDCl_3$) and ^{13}C NMR (151 MHz, $CDCl_3$) spectra for compound **6c**.

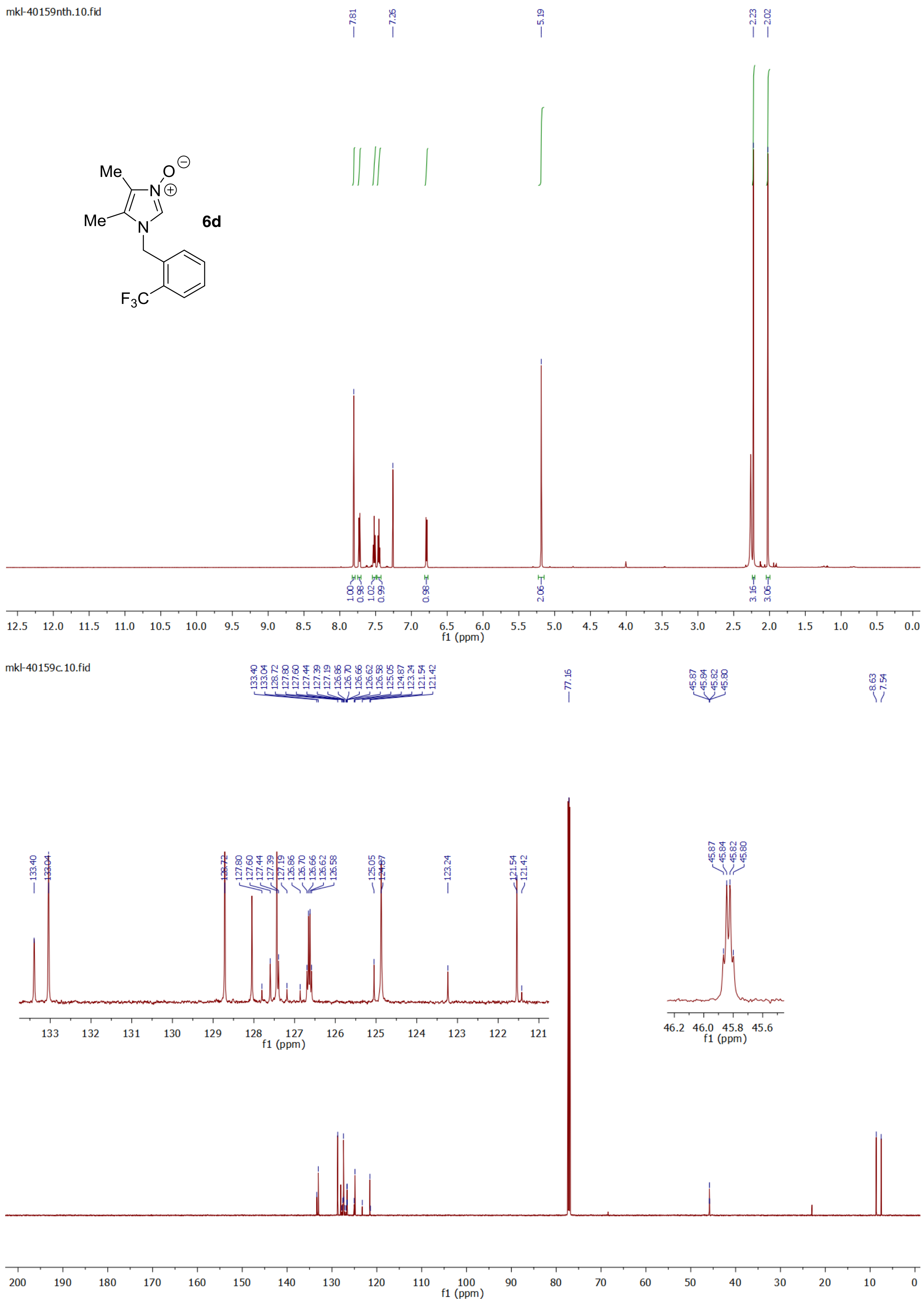

Figure S9. ^{1}H NMR (600 MHz, $CDCl_3$) and ^{13}C NMR (151 MHz, $CDCl_3$) spectra for compound **6d**.

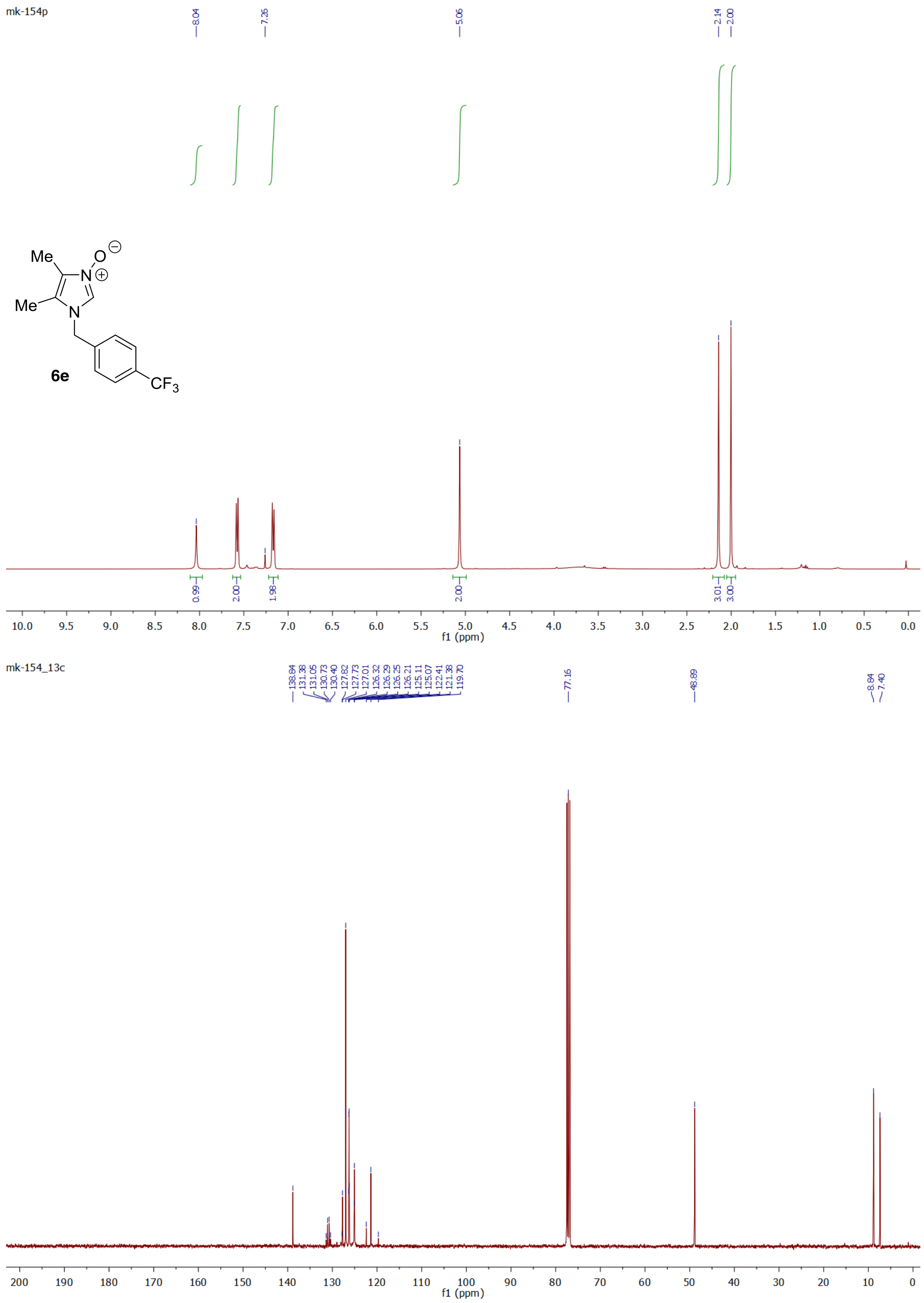

Figure S10. ^{1}H NMR (600 MHz, $CDCl_3$) and ^{13}C NMR (151 MHz, $CDCl_3$) spectra for compound **6e**.

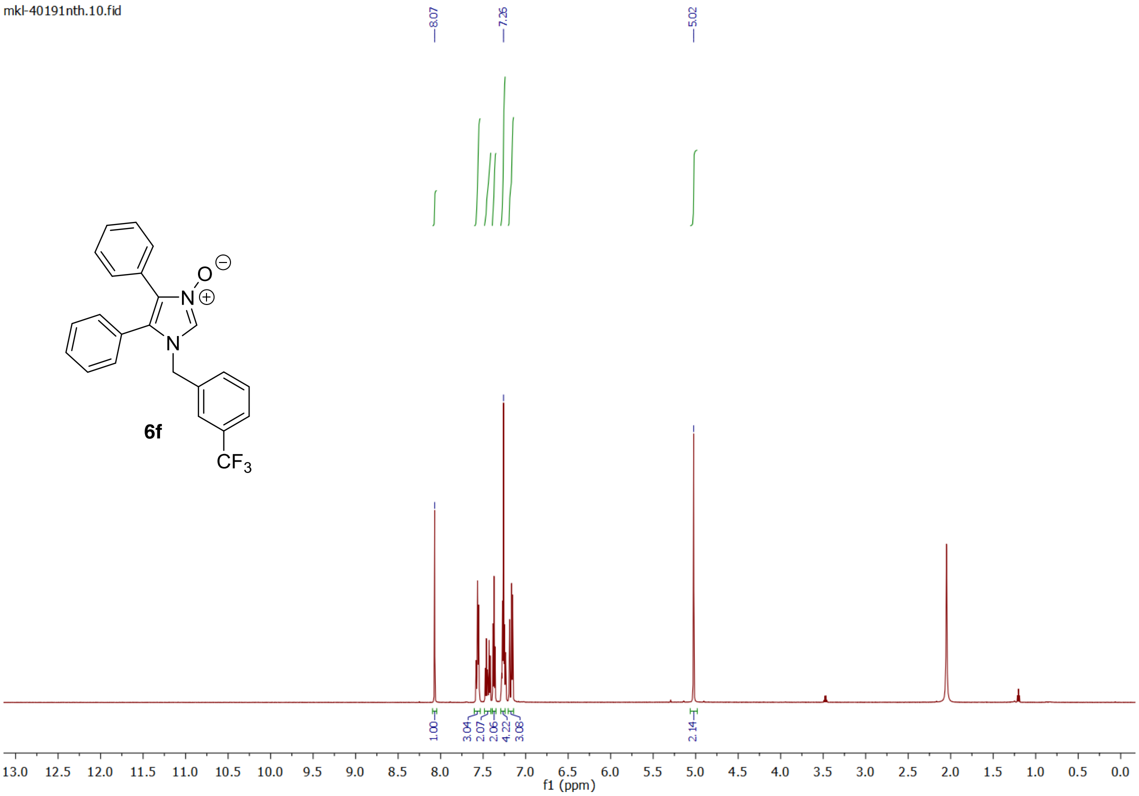

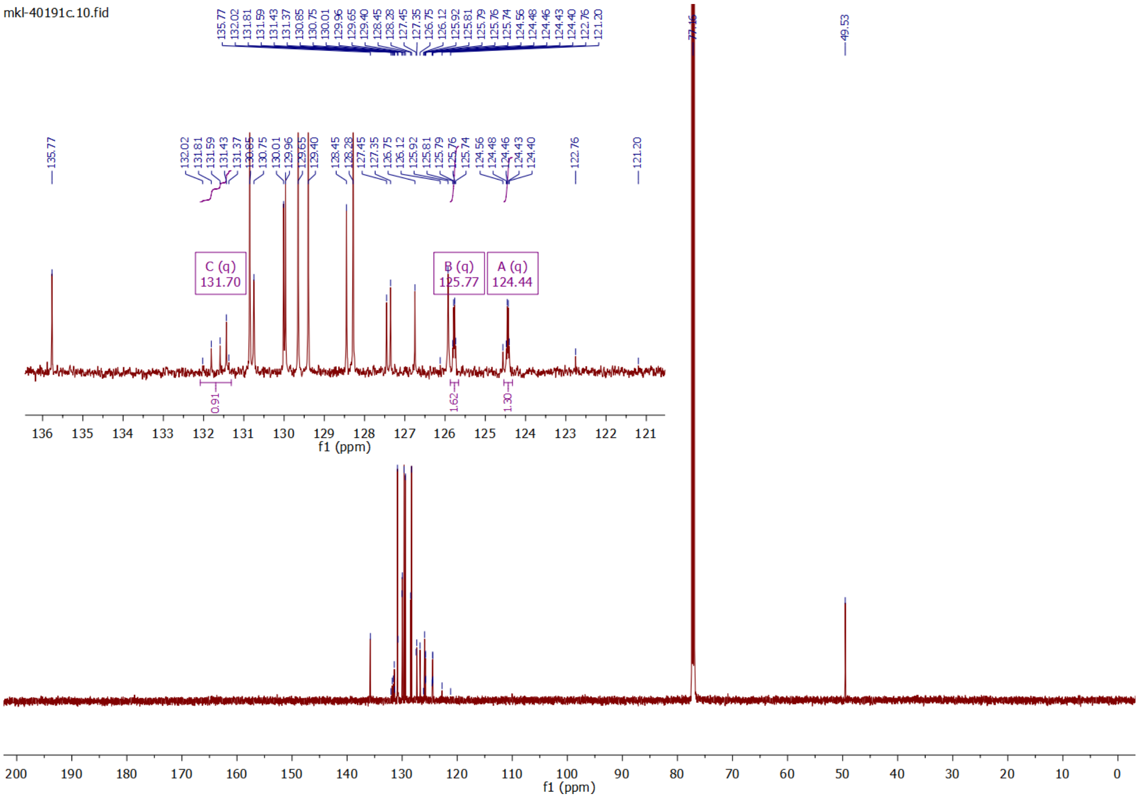

Figure S11. ^{1}H NMR (600 MHz, $CDCl_3$) and ^{13}C NMR (151 MHz, $CDCl_3$) spectra for compound **6f**.

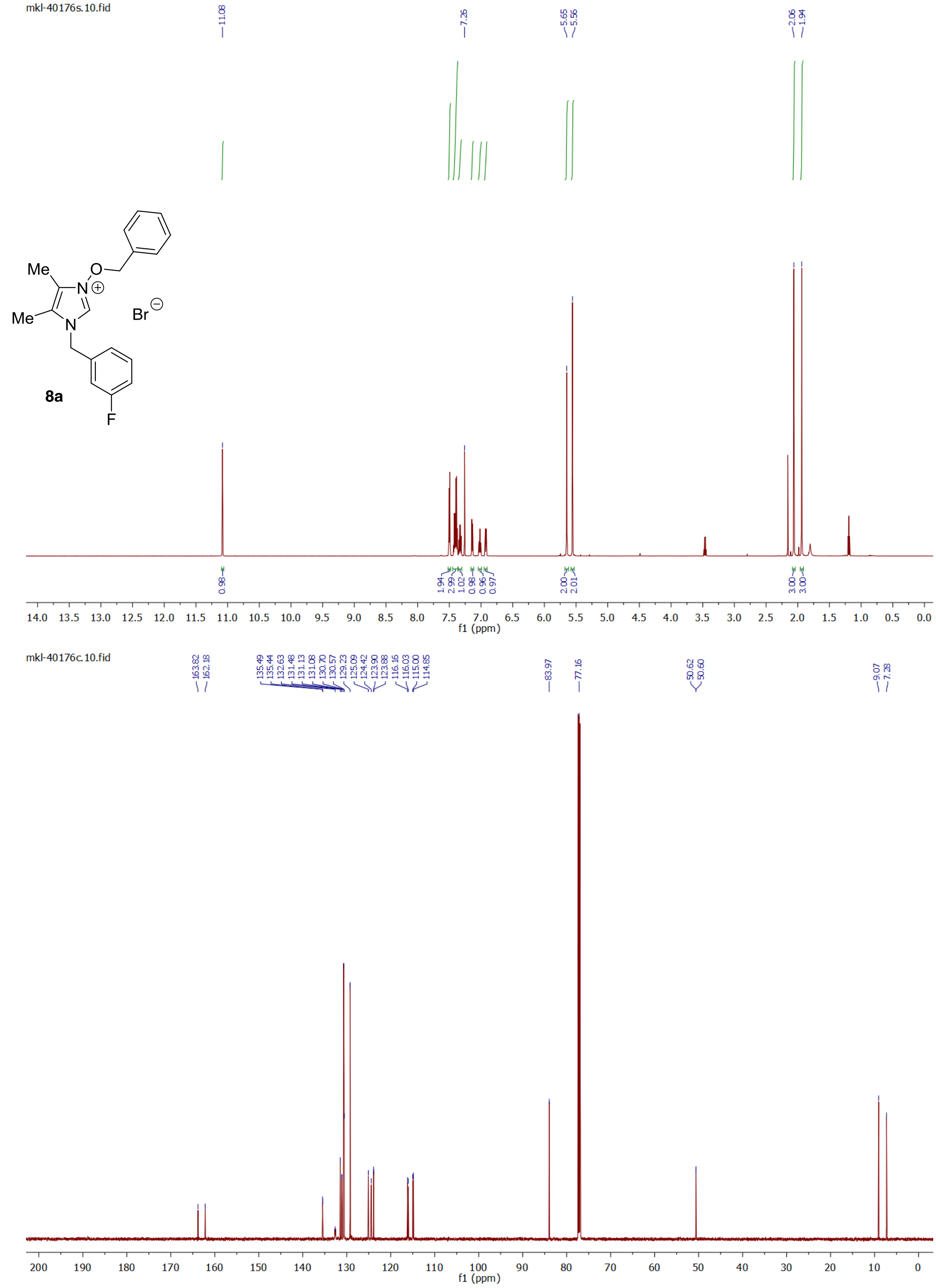

Figure S12. ^{1}H NMR (600 MHz, $CDCl_3$) and ^{13}C NMR (151 MHz, $CDCl_3$) spectra for compound **8a**.

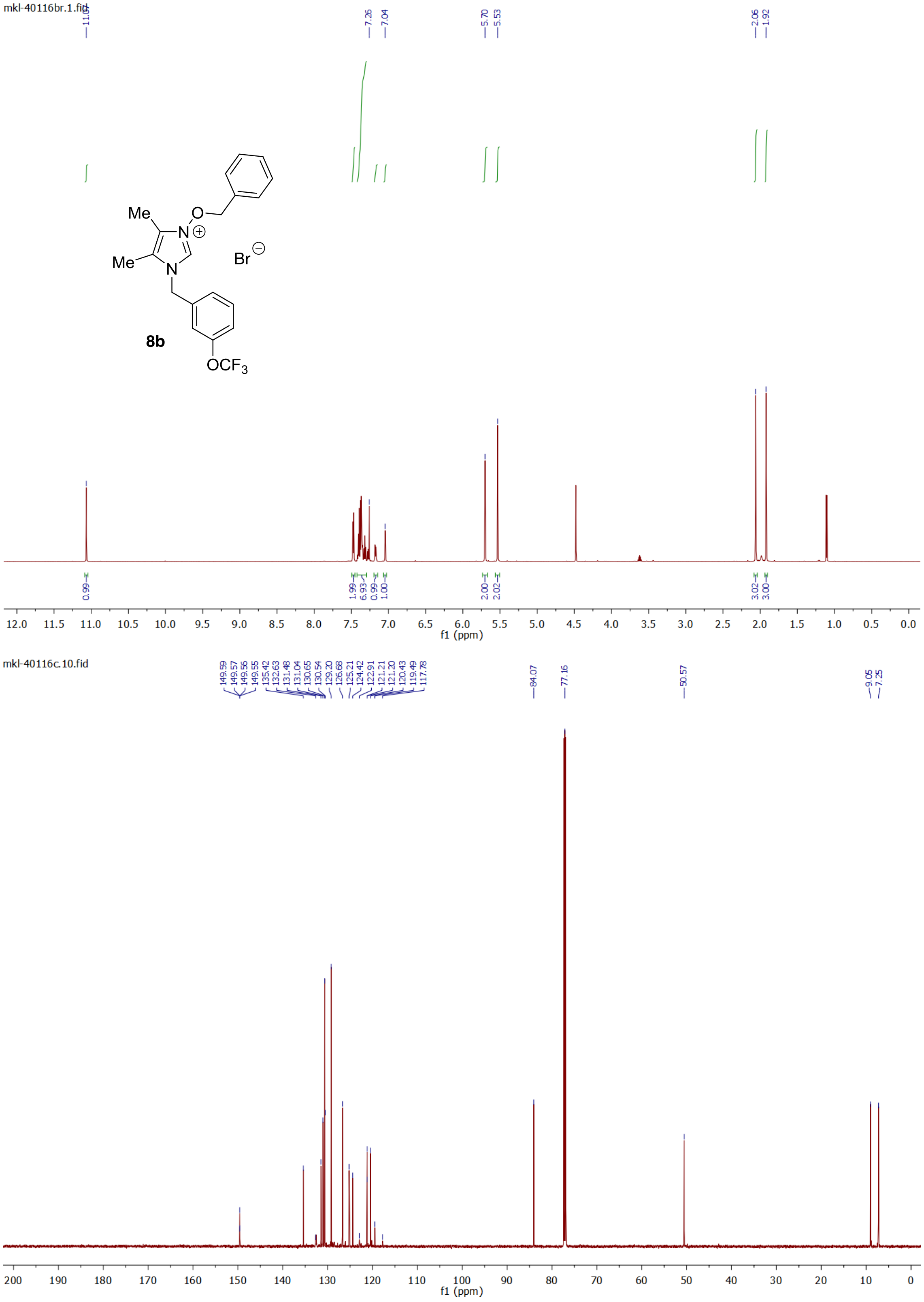

Figure S13. ^{1}H NMR (600 MHz, $CDCl_3$) and ^{13}C NMR (151 MHz, $CDCl_3$) spectra for compound **8b**.

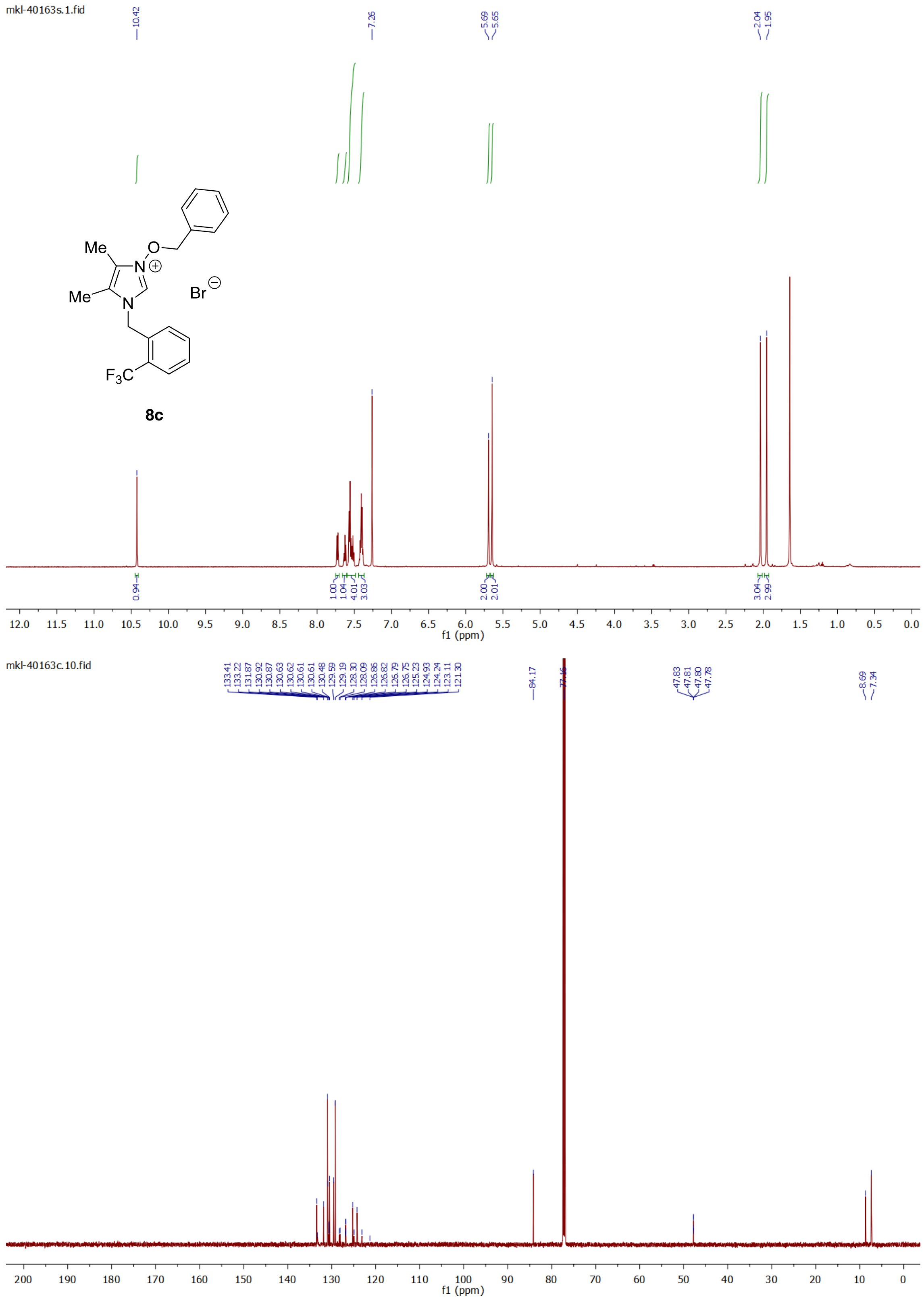

Figure S14. ^{1}H NMR (600 MHz, $CDCl_3$) and ^{13}C NMR (151 MHz, $CDCl_3$) spectra for compound **8c**.

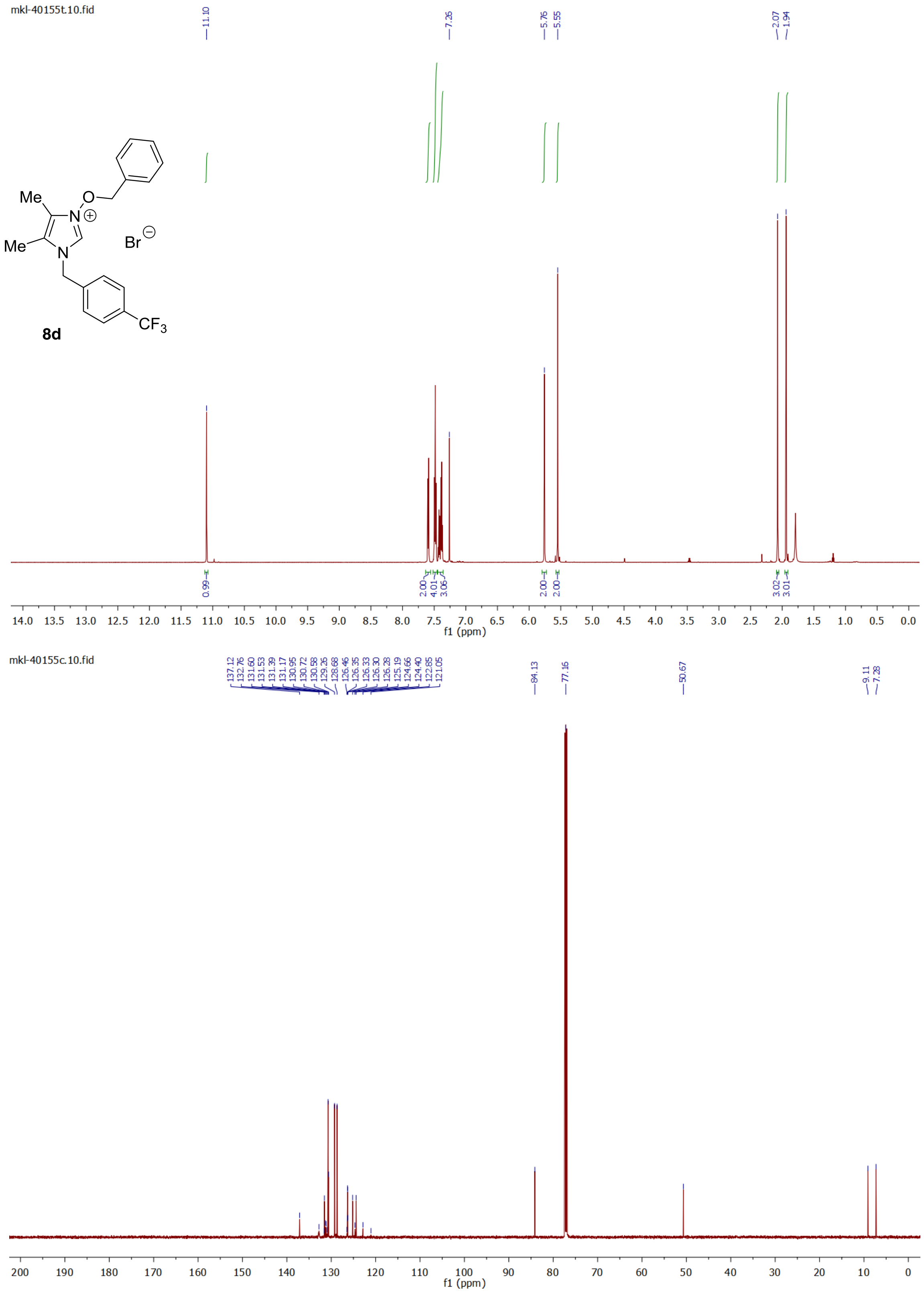

Figure S15. ^{1}H NMR (600 MHz, $CDCl_3$) and ^{13}C NMR (151 MHz, $CDCl_3$) spectra for compound **8d**.

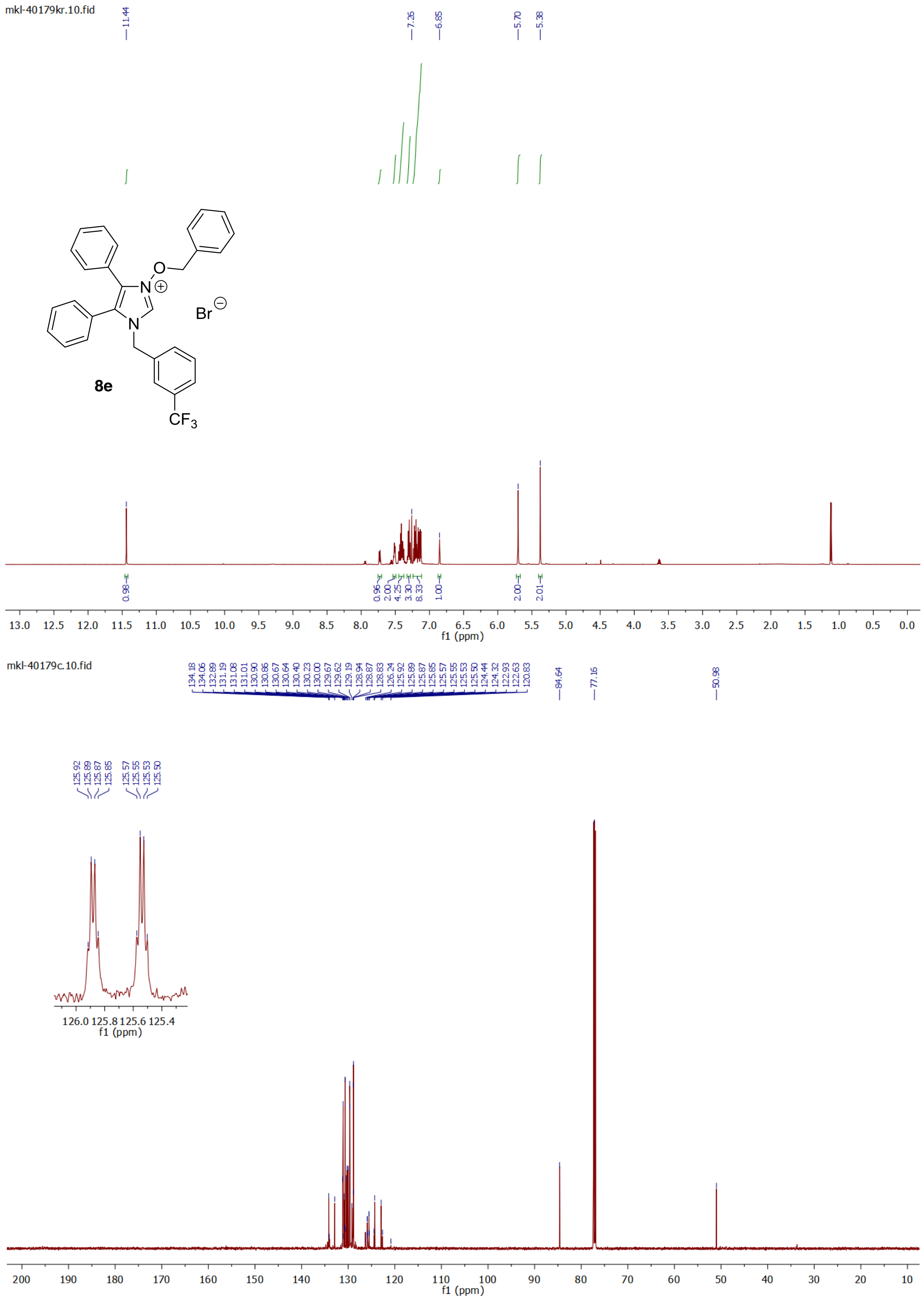

Figure S16. ^{1}H NMR (600 MHz, $CDCl_3$) and ^{13}C NMR (151 MHz, $CDCl_3$) spectra for compound **8e**.

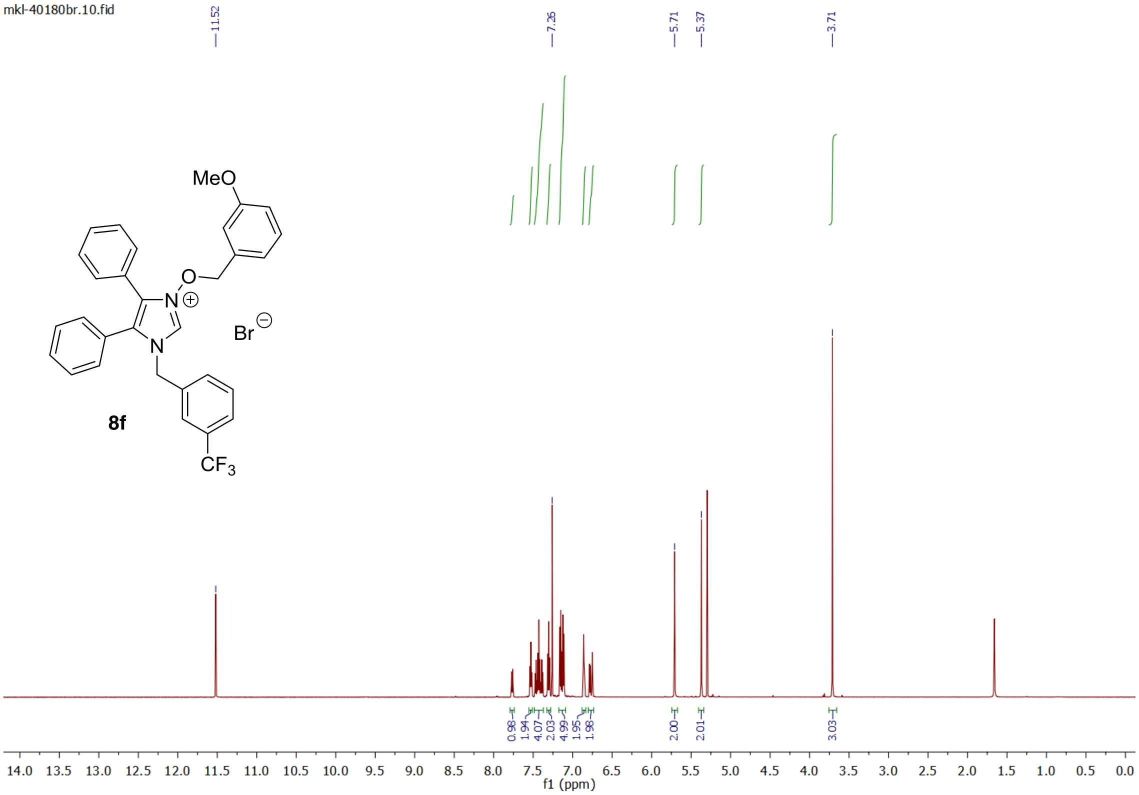

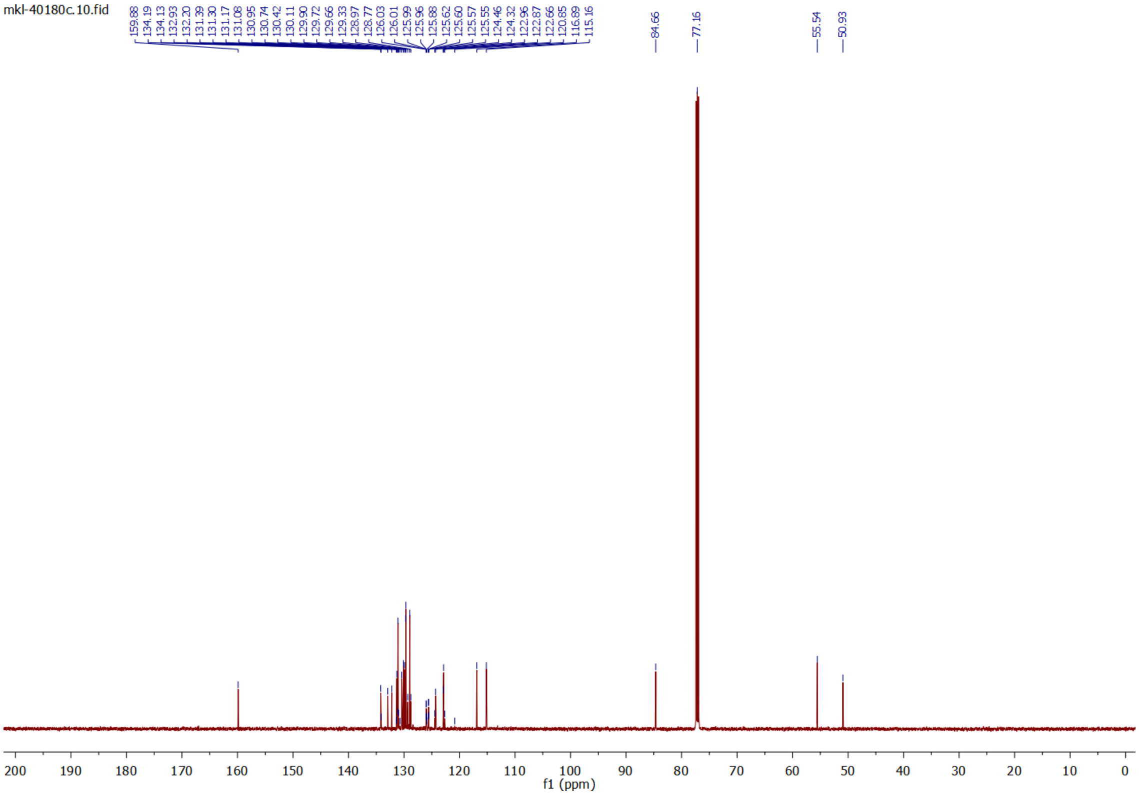

Figure S17. ^{1}H NMR (600 MHz, $CDCl_3$) and ^{13}C NMR (151 MHz, $CDCl_3$) spectra for compound **8f**.

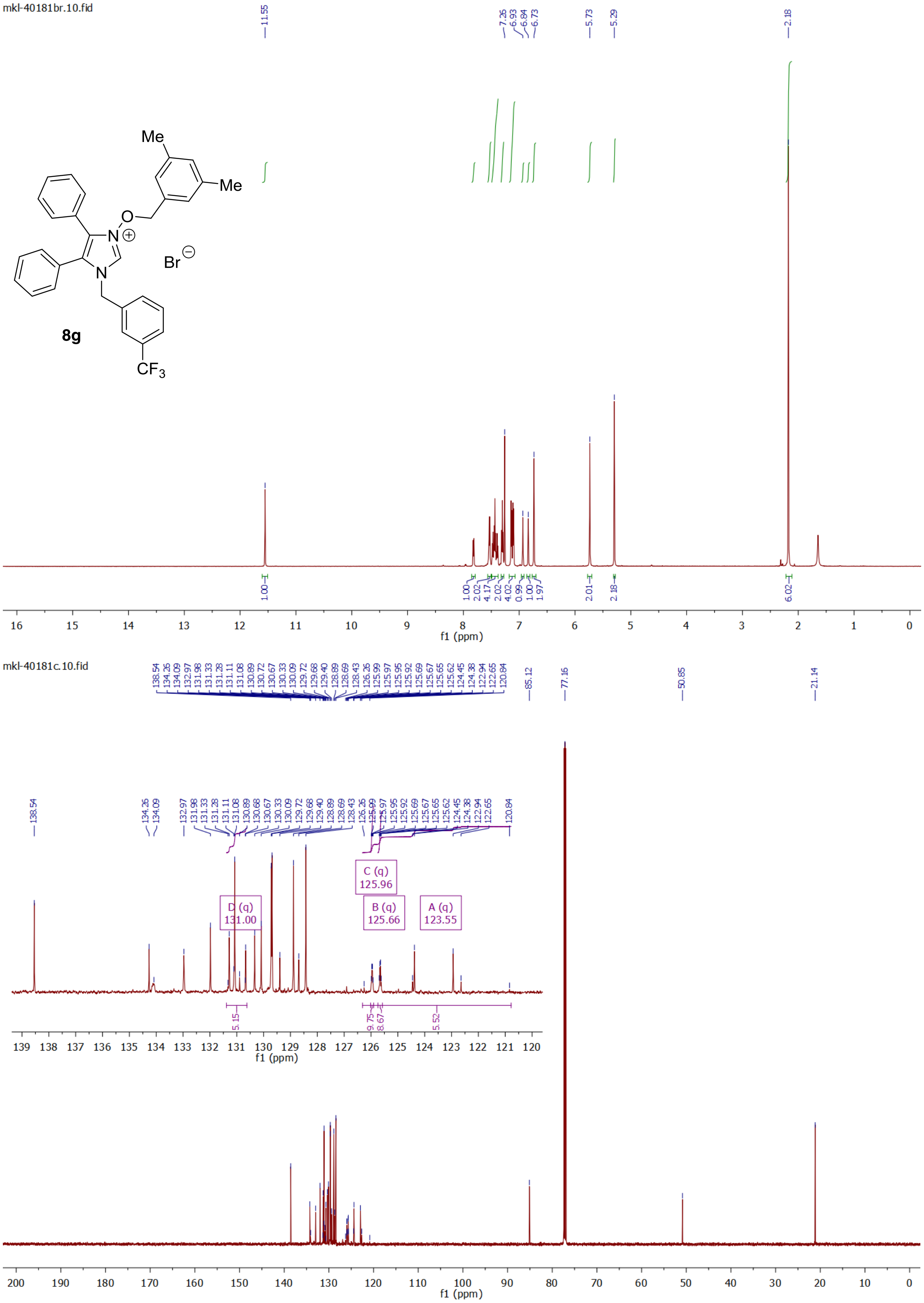

Figure S18. ^{1}H NMR (600 MHz, $CDCl_3$) and ^{13}C NMR (151 MHz, $CDCl_3$) spectra for compound **8g**.

3. Supplementary biological results

The results on cytotoxicity analysis and antiviral screening for lepidilines A and C (**1a** and **1c**), their hexafluorophosphates (**1a[PF$_6$]**, **1c[PF$_6$]**) and selected known structural analogues (**9b[PF$_6$]**, **9c[PF$_6$]**, **10a-10d**) [1] as well as for Arduengo-type imidazolium salt **2a** [2] and its bis-oxo-adamantan-1-yl derivative **2b** [3] are collected in Tables S1-S4.

Compounds demonstrating cell viability ≥ 50% determined in both, cytotoxicity screening (Table S1), as well as antiviral screening (Table S3) were selected for further, extended studies resulting in CC_{50} and IC_{50} parameters calculations. Results of this studies are presented in Table S2 (cytotoxicity studies) and Table S4 (antiviral studies).

Table S1. Results of cytotoxicity screening on Vero, LLC-MK2, NCTC clone 929, MRC-5 and HeLa cells.

Compound	Cell line (viability %)				
	**Vero**	**LLC-MK2**	**NCTC clone 929**	**MRC-5**	**HeLa**
**1a**	90.5	76.3	86.0	90.2	43.0
**1c**	90.7	78.4	8.8	76.7	8.9
**1a[PF$_6$]**	89.0	90.3	56.2	78.6	23.4
**1c[PF$_6$]**	91.3	55.6	8.5	76.9	11.4
**9b[PF$_6$]**	6.7	8.0	4.1	12.8	6.8
**9c[PF$_6$]**	6.6	7.6	6.5	12.6	7.1
**10a**	83.8	64.9	93.8	74.3	61.1
**10b**	29.1	9.1	59.0	100.1	13.3
**10c**	11.4	15.8	35.5	73.8	7.8
**10d**	95.8	53.6	91.5	96.8	74.0
**2a**	7.9	7.8	8.9	73.7	23.0
**2b**	15.2	7.8	6.6	12.7	7.5

Table S2. Cytotoxicity assay in the range of 0.1-1000 μM for compounds demonstrating cell viability ≥ 50% determined in both, cytotoxicity, as well as antiviral screening (selected compounds on LLC-MK2, NCTC clone 929 and HeLa cells).

Compound	Cell line (CC_{50} μM)		
	**LLC-MK2**	**NCTC clone 929**	**HeLa**
**1a**	67.67 ± 2.52	NA	NA
**1c**	28.33 ± 5.86	NA	NA
**1a[PF$_6$]**	32.67 ± 6.81	NA	NA
**1c[PF$_6$]**	75.33 ± 5.51	NA	NA
**10a**	16.17 ± 3.75	46.00 ± 6.93	16.90 ± 2.19
**10d**	NA	NA	318.33 ± 47.52

NA - not applicable, cell viability < 50%

Table S3. Results of antiviral screening against HSV-1, HPIV-3, EMCV, HCMV and AdV5.

Compound	Cell line (viability %)				
	HSV-1	HPIV-3	EMCV	HCMV	AdV5
**1a**	48.0	86.0	7.4	1.9	45.8
**1c**	18.6	90.4	7.7	0.2	17.9
**1a[PF$_6$]**	17.5	62.4	7.8	4.5	15.3
**1c[PF$_6$]**	23.3	91.1	7.9	4.5	17.1
**9b[PF$_6$]**	11.7	12.0	9.3	6.3	13.9
**9c[PF$_6$]**	13.3	12.8	9.4	1.9	14.4
**10a**	29.7	69.2	79.6	3.6	62.0
**10b**	16.5	7.9	8.1	1.9	39.5
**10c**	14.7	10.9	8.5	7.1	16.0
**10d**	14.6	10.6	8.5	1.9	68.0
**2a**	13.9	8.3	7.1	9.7	50.2
**2b**	14.1	8.7	9.6	2.8	17.1

Table S4. Antiviral assay in the range of 0.1-1000 µM for compounds demonstrating cell viability ≥ 50% determined in both, cytotoxicity, as well as antiviral screening (selected compounds on HPIV-3, EMCV and AdV5).

Compound	IC_{50} (µM)		
	HPIV-3	EMCV	AdV5
**1a**	>67.67	NA	NA
**1c**	>28.33	NA	NA
**1a[PF$_6$]**	>32.67	NA	NA
**1c[PF$_6$]**	>75.33	NA	NA
**10a**	>16.17	>46.00	>16.90
**10d**	NA	NA	>318.33

NA - not applicable, cell viability < 50%

4. References

1. Mlostoń, G.; Kowalczyk, M.; Celeda, M.; Gach-Janczak, K.; Janecka, A.; Jasiński, M. Synthesis and cytotoxic activity of lepidilines A-D: comparison with some 4,5-diphenyl analogues and related imidazole-2-thiones. *J. Nat. Prod.* **2021**, *84*, 3071–3079.
2. Arduengo III, A.J.; Harlow, R.L.; Kline, M. A stable crystalline carbene. *J. Am. Chem. Soc.* **1991**, *113*, 361–363.
3. Mlostoń, G.; Celeda, M.; Urbaniak, K.; Jasiński, M.; Bakhonsky, V.; Schreiner, P.R.; Heimgartner, H. Synthesis and selected transformations of 2-unsubstituted 1-(adamantyloxy)imidazole 3-oxides: straightforward access to non-symmetric 1,3-dialkoxyimidazolium salts. *Beilstein J. Org. Chem.* **2019**, *15*, 497–505.
